# The banality of counterterrorism “after, after 9/11”? Perspectives on the Prevent duty from the UK health care sector

**DOI:** 10.1080/17539153.2018.1494123

**Published:** 2018-07-17

**Authors:** Charlotte Heath-Kelly, Erzsébet Strausz

**Affiliations:** a Politics and International Studies, University of Warwick, Coventry, United Kingdom

**Keywords:** Prevent strategy, safeguarding, NHS, health care, radicalisation

## Abstract

Since 2015, the UK healthcare sector sector has (along with education and social care) been responsibilised for noticing signs of radicalisation and reporting patients to the Prevent programme. The Prevent Duty frames the integration of healthcare professionals into the UK’s counterterrorism effort as the banal extension of safeguarding. But safeguarding has previously been framed as the protection of children, and adults with care and support needs, from abuse. This article explores the legitimacy of situating Prevent within safeguarding through interviews with safeguarding experts in six National Health Service (NHS) Trusts and Clinical Commissioning Groups. It also describes the factors which NHS staff identified as indicators of radicalisation – data which was obtained from an online questionnaire completed by 329 health care professionals. The article argues that the “after, after 9/11” era is not radically distinct from earlier periods of counterterrorism but does contain novel features, such as the performance of anticipatory counterterrorism under the rubric of welfare and care.

## Introduction

The question “is there an after, after 9/11?” forces us to address whether there has been any return from the exceptionalised, emergency frame which dominated international politics in the aftermath of 9/11. At face value, the direction of travel in Europe and North America is unchanged. The immediate legislative responses to international terrorism of the twenty-first century – the Contest Strategy (UK) and the Patriot Act (USA) – have not been repealed, and the technological advance of counterterrorism surveillance continues unabated.

However, this article argues that Countering Violent Extremism (CVE) policies do represent a distinctive development in the post-9/11 counterterrorism repertoire. CVE frameworks are now employed across the globe by actors such as the United Nations, the European Union as well as individual nation states (Kundnani and Hayes 2018). Their unique selling point is the prevention of terrorism through interventions in “radicalisation processes”, before criminal conspiracy occurs (in the so-called “pre-criminal space”). Here, the resources usually associated with social welfare policies (like mentoring, housing and education) are reframed as terrorism prevention assets (Home Office 2017, 2018). In contradistinction to the emergency criminal justice measures of the immediate 9/11 era, this article focuses on the banality of the Prevent Duty in the UK where social care, education and health care workers are now engaged in the prevention of terrorism (Ragazzi 2017).

The Counter-Terrorism and Security Act of 2015 placed a statutory duty on specified authorities within the UK’s public sector to have “due regard to the need to prevent people from being drawn into terrorism” (Home Office 2015: 2). In practice, this has meant that schools, colleges, universities, prisons, social services, National Health Service (NHS) trusts, NHS Clinical Commissioning Groups^1^ (CCGs) and private health care providers on the NHS standard contract now perform counterterrorism – under the rubric of safeguarding. These providers deliver Prevent-related training to their staff, promote the importance of the Prevent Duty to their staff, and process Prevent referrals made about colleagues, students, patients and clients.

The use of health and education sectors to support counterterrorism is unique to England, Wales and Scotland. The Prevent Strategy has not even been rolled out to the entire UK (given the specific provisions of Good Friday Agreement, the Prevent Strategy does not apply in Northern Ireland). While some European nations utilise community policing and prison services to identify and deter potential radicals, it is much rarer for the non-judicial sectors to be involved in counter-radicalisation. The Netherlands is an exception and has begun encouraging youth workers and educators to promote counter-radicalisation^2^ agendas. But Britain is alone in making its educational, health and social care sectors legally responsible for counter-radicalisation.

In this unique context of policy implementation, this article explores how counter-radicalisation has been integrated into the health care sector. The authors conducted a 12-month pilot study, funded by the Wellcome Trust, which evaluated the performance of Prevent Duty safeguarding by NHS trusts and CCGs in the Midlands region of England.^3^ Using expert interviews with NHS safeguarding professionals, discourse analysis of the Workshop to Raise Awareness of Prevent (WRAP) training DVD and a survey of staff attitudes (*N* = 329) towards the Prevent Duty, we explored how Prevent is performed within the NHS’ safeguarding remit.

Our study showed that NHS staff are, on the whole, accepting of their new responsibilities under the Prevent Duty and comfortable with the training provided. However, our survey also revealed that staff knowledge of specific “radicalisation indicators” is drawn not from official training but from popular culture representations of ISIS and radicalism. WRAP training encourages staff to intuit the presence of radicalisation in staff and patients, rather than providing details of explicit “signs” to be reported. We found that this has led NHS staff to rely on the media for their understandings of terrorism and radicalisation. Furthermore, despite the popular acceptance of the Prevent Duty as a form of safeguarding, our expert interviewees unanimously commented on the imperfect fit between the Prevent Duty and other forms of safeguarding expected of the NHS.

Unlike broader safeguarding protocols, Prevent safeguarding is not restricted to adults with “care and support needs”. NHS England therefore defines all citizens as potentially vulnerable to radicalisers and opens the possibility for care practitioners to intervene upon their lives. This extension of interventions across the entire population (rather than limiting them to those persons with care and support needs) creates a tension with the liberal foundations of British political life. A distinctive feature of counterterrorism “after, after 9/11” is, then, that care can be positioned as an anti-terrorism measure – and counterterrorism as a care intervention; such is the normalisation of the counterterrorism within society “after, after 9/11”.

## Prevent in the NHS: academic studies

The majority of academic literature on Prevent in UK public services addresses the performance of the duty in the particular contexts of education (Choudhury 2017; Miah 2013; Thomas 2016), youth work (Lynch 2013; McDonald 2011) and social work (McKendrick and Finch 2017). Studies exploring the role of the health care sector in Prevent are beginning to emerge, but at a slower pace. Research access to the NHS can be a lengthy and complicated procedure involving a primary application to the Health Research Authority, unfamiliar terminologies (social scientific research is often not recognised as “research” but as a “service evaluation”) and secondary research access applications to each local NHS Trust's Research and Innovation (R&I) department. These difficulties render studies of Prevent in the NHS slower to develop. To date, literature on the Prevent Duty in health care can be divided into two streams: debates in forensic psychiatry about the scientific reliability of extremism risk scoring tools and social scientific research into the NHS as a counter-radicalisation actor. This literature review focuses only on the latter.

In the field of Psychiatry, David Goldberg, Jadhav, and Younis (2017) have recently problematised the use of the term “pre-criminal space” in NHS England Prevent Training and Competencies documentation. To foster acceptance in the health care sector, the term situates the positioning of Prevent as a safeguarding and protection measure. Intervening to protect people in the “pre-criminal” space emphasises the separation of Prevent safeguarding from judicial measures. As Goldberg et al. rightly point out, the obscure term “pre-criminal space” does not appear in Home Office Prevent policies nor in any other NHS safeguarding policy: in policy terms, it is unique to NHS England’s Prevent Guidance. They explore the etymology and usage of “pre-criminal space”, arguing that it operates as both metaphor and analogy – smoothing and bridging the discourses of the health and criminal justice sectors, and persuading NHS professionals to comply with the duty (Goldberg, Jadhav, and Younis 2017).

Other academic research into the Prevent Duty focuses on the mass responsibilisation of health care workers for counterterrorism detection and prevention. Heath-Kelly (2017a, 2017b) has explored how the Prevent Duty expands the application of preventative counterterrorism to the entire population, rather than deploying it upon smaller suspect communities. The training of the 1.3 million NHS workers to report radicalisation demonstrates a transition of security apparatus away from security professionals (such as counterterrorism officers) who possess formal training, towards a far looser type of counterterrorism “expertise”. In the NHS, a 1- or 2-hour training session in front of the “WRAP” DVD qualifies a staff member to detect and report radicalisation. The minimal training for such a sensitive task shows that central government have prioritised large-scale reporting of radicalisation suspicions across the population, rather than targeted and precise counterterrorism measures (Heath-Kelly 2017a, 2017b). Indeed, upon the release of the 2013 Prevent Guidance document “Building Partnerships, Staying Safe”, Director of Nursing Hilary Garratt wrote to all CCG commissioning leads and emphasised that the size of the NHS (which makes 1 million patient contacts every 36 hours), rather than any specific expertise, qualified it as a key partner within the Prevent Strategy (Garratt 2013). Size matters, it seems, in the new Prevent Duty.

Outside these formal studies, various health care professionals have also taken to the pages of academic and professional journals to voice concern, and occasionally support, for the Prevent Duty in the NHS. Derek Summerfield, a consultant at South London and Maudsley NHS Trust, wrote in *BJPsych Bulletin* that compelling medical staff to attend Prevent training is as follows:

a corrosion of the ethics of the doctor-patient relationship, and is to prime us for an activity which is a duplicitous deviation from the medical assessment, advice and treatment that has brought the patient to us. (Summerfield 2016)

Dr Rosemary Rizq has similar concerns about the conflation of health care and protection with the reporting of terrorist deviance. She argues that surveillance and control insidiously invade the consulting room through the Prevent Duty, turning the patient requiring health care into an already-securitised risk, and forbidding the free play of speech so essential for therapy (Rizq 2017).

## Prevent as safeguarding?

This perceived clash between medical ethics and reporting radicalisation suspicions is complicated by the positioning of the Prevent Duty as a safeguarding measure. Central government policy has explicitly presented the Prevent Duty as an additional patient safeguarding measure which entails no extra responsibilities on behalf of clinical and non-clinical staff (Department of Health 2011: 3). Accordingly, NHS England places responsibility for Prevent training and the processing of Prevent referrals with Trust and CCG safeguarding teams. These safeguarding experts provide WRAP training to staff with the Home Office DVD and script, process Prevent queries (filtering out inappropriate referrals), and determine which queries are referred to Local Authorities and the police. Safeguarding discursively and operationally situates Prevent in the NHS.

While safeguarding is more loosely defined as a “protective intervention” in the educational and social care sectors, safeguarding processes in the NHS are tightly defined. Health care safeguarding is designed *to protect those with care and support needs* (like learning disabilities, severe mental health conditions, dementia, drug and alcohol addiction) from abuse, where they cannot protect themselves. They are a necessary societal protection for those with reduced individual capacity or agency. The Care Act was passed in 2014 to legally enforce the safeguarding of vulnerable people, and it states that local authorities must intervene to support adults in cases:

where a local authority has reasonable cause to suspect that an adult in its area (whether or not ordinarily resident there) – (a) *has needs for care and support* (whether or not the authority is meeting any of those needs), (b) *is experiencing, or is at risk of, abuse or neglect, and* (c) as a result of those needs is unable to protect himself or herself against the abuse or neglect or the risk of it. (2) The local authority must make (or cause to be made) whatever enquiries it thinks necessary to enable it to decide whether any action should be taken in the adult’s case. (Care Act 2014, c.23, section 42, emphasis added)

If an adult has care and support needs, faces the risk of abuse *and* cannot protect themselves, then the individual’s agency ceases to be a paramount principle. Rightly, the Local Authority becomes duty bound to intervene in these situations.

In the NHS, safeguarding teams take reports about vulnerable patients who are at risk of abuse (physical, sexual, financial) and liaise with the Local Authority, and that patient, to put a care package in place. But under the Prevent Duty, safeguarding protocols are activated in cases where no care and support need exists. In Prevent protocols, the explicit provision regarding “care and support needs” in the Care Act is watered down to a suggestion of “vulnerabilities” – creating concerns that the presumption of adult agency has been side-lined.

Home Office and NHS England policy guidance presents radicalisation as a process of abusive exploitation performed upon “vulnerable” persons. But vulnerability, under the Prevent Duty guidance protocols, is no longer a formal state of reduced capacity (like care and support needs); rather, “vulnerability” is extended to potentially cover the entire population. Anyone, with or without care needs, can be the subject of a Prevent safeguarding inquiry. How is this interference in a person’s agency justified, before the criminal threshold is reached? One’s vulnerability (and the need for safeguarding intervention) is constituted through a circular argument: simply by being associated with a radicalisation referral, a person *must already have been vulnerable* to extremist influence. The assumption plays out that no person with full capacity, or in control of their life, would support political extremism or terrorism – thus any presumed sympathy denotes reduced capacity or “vulnerability”.

While any person can be subject to a Prevent safeguarding referral, regardless of their formal capacity level, the Department of Health does provide some guidance on “factors which put people at risk of exploitation by radicalisers”. Worryingly, these racialise “vulnerability” to extremism. The factors emphasise the migration status of an individual as a point of vulnerability to extremism, their “traditional” family life (in a context where “traditional” acts as a synonym for people of colour), and their “religious/cultural heritage”. For example, the first factor highlighted by the Department of Health as indicating radicalisation vulnerability is “identity crisis”, but this is characterised as follows:

Adolescents/vulnerable adults who are exploring issues of identity can feel both distant from their parents/family and cultural and religious heritage, and uncomfortable with their place in society around them. (Department of Health 2011: 10)

Identity crisis seems to be associated here with second- or third-generation immigrants, positioned between cultures. Apparently “identity crisis” is not a potential experience that anyone could experience, only specific and racialized groups.

The second factor in the Department of Health’s list of radicalisation vulnerability factors is “personal crisis” (characterised as the “isolation of the vulnerable individual from the *traditional* certainties of family life” [emphasis added]). Following on from the first racialised indicator, this invocation of “traditional family life” reads like a synonym for racialised groups in society. The third factor in vulnerability relates to an individual’s “personal circumstances”. We all have “personal circumstances”, but these are characterised by the Department of Health in terms of cultural, religious and raced identities leading towards radicalisation vulnerability:

Personal Circumstances: The experience of migration, local tensions or events affecting families in countries of origin may contribute to alienation from UK values and a decision to cause harm to symbols of the community or state. (Department of Health 2011: 10)

Finally, unemployment/underemployment and criminality are listed (without racialised undertones) as factors which may make a person vulnerable to radicalisation (Department of Health 2011: 10).

Without a clinical evidence base or NICE guidance, these factors replace the formal care and support needs which are central to other forms of NHS safeguarding. These “factors” fudge the centrality of care and support needs to safeguarding (Care Act 2014, c.23, section 42) by repeatedly invoking the “vulnerability” of a person experiencing disenfranchisement or ennui, but provide no other basis for the interruption of their agency by a safeguarding action.

Without care and support needs, an adult should be entitled to live uninterrupted by the state unless they request support and assistance. Indeed, NHS safeguarding policy enshrines the principle of agency, in all cases apart from where “coercion and undue influence” might apply:

Adults have a legal right to make their own decisions, even if they are unwise, as long as they have capacity to make that decision and are free from coercion or undue influence. (NHS England 2015: 16)

Given the usual presumption of agency for adults without care and support needs, Prevent in the NHS stretches normal safeguarding customs and protocols to their limit. The “coercion and undue influence” usually understood to constitute impaired agency are severe situational constraints. For example, people experiencing domestic violence or human trafficking can receive safeguarding assistance despite not necessarily possessing “care and support needs” – because they are experiencing significant coercion and abuse from which they cannot reasonably be expected to protect themselves.

But is it legitimate for Prevent to equate the coercion experienced by human trafficking and domestic violence victims, trapped within appalling situations and experiencing dramatic constraints upon their agency, to those engaging with extreme political opinions and those who voice them? Given the potentially dramatic contrast, it would be far more appropriate to presume that individuals retain agency and can engage with political ideas freely. While the intention of safeguarding practitioners is to keep people safe from travelling to war zones or becoming involved in terrorism, the Prevent Duty involves a significant departure from the presumption of adult agency in cases where there is often no cognitive or coercive impediment.

In the next section, we highlight the testimonies of safeguarding leads within NHS trusts and CCG’s on the imperfect fit between Prevent and Safeguarding. These safeguarding experts experienced significant dissonance between their desire to protect potential victims of radicalisers, and their knowledge that safeguarding interventions explicitly require the recipient to have “care and support needs” to justify the intervention upon agency.

## NHS safeguarding experts on the prevent strategy

Our interviews with safeguarding experts shed light on some of the key features of Prevent’s uneasy situation within existing safeguarding infrastructure. Most of the safeguarding experts we interviewed held conflicting views and beliefs with regards to the place, function and broader implications of Prevent referrals made by NHS staff, even if they had come to accept, endorse or re-appropriate the Prevent Duty. These dissonances in how safeguarding experts see their role reveal important tensions and concerns that arise when a counterterrorism reporting structure is embedded within an otherwise evidence-based medical culture.

Normal safeguarding referrals utilise a transparent process which is subject to audit and clinical governance. They follow an approach of person-centred care that presupposes the person’s consent and in line with general professional guidelines in the NHS, foregrounds patient choice. However, the tension between Prevent and the medical duty to provide care was immediately noted by some of our expert interviewees. One General Practitioner (GP) we spoke to noted that with Prevent the very object and objective of protection changes: “When you do safeguarding, the person sat in front of you is your main concern because you’re trying to protect that person. Whereas with this, you’re protecting the state from that person” (Consultant Psychiatrist B and GP 2, 2017). This underlying shift makes Prevent a controversial subject within the NHS, but also demonstrates an imperfect fit with existing safeguarding structures which necessitates a constant negotiation of the Prevent Duty by health care professionals.

Upon the introduction of Prevent to the NHS, a CCG Prevent Lead (responsible for giving the Prevent training) recalled that the policy “didn’t originally sit very well with [them] or a lot of [their] team” (CCG Prevent Lead B, 2017). These concerns reflect the problematic situation of Prevent within NHS safeguarding. Prevent aims to orchestrate a new way of “seeing” and performing safeguarding by cultivating an “awareness” for vulnerable people being at risk of radicalisation. Prevent sits in tension with the approaches, principles and definitions of safeguarding under the Care Act. These tensions become apparent when one attempts to understand the type of “abuse” upon which Prevent intervenes. Normal safeguarding processes exist to protect vulnerable people from financial, physical and sexual abuse, where they cannot protect themselves. For Prevent interventions, the closest fit would be protection from psychological abuse, qua domestic violence safeguarding. But even this fit is imperfect. One expert practitioner of Prevent safeguarding understood radicalisation as a “type of grooming” evident when “people are being harassed, they’re being groomed, they may not have any control over what they’re being drawn into […]. Particularly if they’re very vulnerable and they don’t recognise it” (Safeguarding Expert P, 2017).

Yet a different Prevent trainer pointed out to us that the notion of “vulnerability” is used and talked about very differently between the safeguarding and Prevent contexts. Instead of a focus on “care and support needs”, what is emphasised is a “complex set of grievances” related to ideology, exclusion and identity. For example,

Prevent, to me, is about finding people who are at the cusp in their lives of not having anything else other than somebody saying, “why don’t you do this?” […] And for a young Muslim child growing up, I suppose, who has a strong family bond and then suddenly is an isolated teenager, racially abused perhaps, can’t find work, you’re going to start connecting with things that perhaps, you know, you see and hear and read, and to deal with your own feelings of anger: “oh, why haven’t I got a job? Why am I in this position?” And that’s who Prevent is meant to protect, but the fact that it sits under Safeguarding, I don’t think it sits comfortably with Safeguarding. (CCG Safeguarding Expert C, 2017)

Here, the type of abuse that NHS staff are duty-bound to protect against seems to be exclusion, rather than the existing typologies of abuse within safeguarding regimes.

The introduction of the Prevent Duty as a form of safeguarding protection has led to professional dissonance. During our interviews with safeguarding experts, it was common for them to first make the case that Prevent fits well within safeguarding – on the basis that the professional intuition already developed within safeguarding practice is an appropriate tool for detecting radicalisation. In this regard, the Prevent Duty requirements made sense to staff. Safeguarding professionals were described as having a particular nose for detecting the unfamiliar, understood as a “gut instinct that there’s something, but I don’t know what” (Safeguarding Experts F and J, 2017). But interviewees would then draw out some differences in how this intuition is utilised within Prevent. The safeguarding remit, they acknowledged, is now extended beyond people with care and support needs. Furthermore, in normal safeguarding, the emphasis is on “making the person immediately safe,” which is not the case in Prevent (Ibid).

However, the ways in which Prevent stretches beyond the scope of safeguarding was (despite these inconsistencies) often seen in a positive light. The point was made that in actual practice, normal safeguarding *also* extends beyond the strict definitions of the Care Act. Safeguarding experts F and J framed this work as a banal type of intervention upon society: “like any one of us […] have been a victim of something, but haven’t got care and support needs, but still need some signposting” (Ibid). In this sense, they argue that “although it’s different, I suppose, [Prevent] is not that different, because some of what we do sits in and out of safeguarding as it sits under the Care Act” (Ibid). Here, Prevent was framed as “early help,” before something “reaches a critical point”. These safeguarding experts saw the Prevent Duty as complementary to the Care Act as “we’re not always working completely in line with the Care Act, because there’s more to it, and we will try and prevent things before they get to that point” (Ibid).

Other safeguarding experts also made similar rhetorical moves which positioned Prevent within the safeguarding regime and existing practice. It is “almost parental care” for an individual rather than being ‘explicit and saying, actually, this is an act … this is dangerous, this is potentially dangerous (CCG Safeguarding Expert C, 2017). This is interesting, because it shows how the provisions of the Care Act and the Prevent Duty have been appropriated and renegotiated by health care professionals. Prevent’s vagueness on “vulnerability” has been re-appropriated to redirect attention to the needs of, and rectifying the exclusion of, the person. One interviewee used it as a “pathway” – something like a personal care-centred corrective to the Care Act – in order to secure resources for clients who would otherwise have fallen through the safety net of social care (Ibid).

Yet, this negotiation of ambiguities is also practised by Central Government, who have manufactured an overlap between NHS safeguarding and the anticipatory surveillance of potential radicals. Unlike normal safeguarding, Prevent referrals from the NHS are fed directly to the police as a matter of routine. In regular clinical practice, collaboration with the police follows established procedures: in cases where clear threats to personal safety are voiced, the police are called first and concerns about health come second. But Prevent operates before this threshold of explicit threat and involves the police as a matter of course. If a health care professional makes a Prevent referral, it is first screened by the safeguarding team before those cases deemed Prevent-worthy progress to the stage of police disambiguation (Home Office 2017: 5–6). The health care professional as well as the safeguarding team screen for signs of “radicalisation” that stop short of threats to personal safety, before involving the police. But what kind of expertise informs this professional judgement?

Trainers and safeguarding teams are not experts in counterterrorism: they receive the same WRAP training as those they train, while also receiving occasional briefings from the Regional Prevent Coordinator. Interviewees confirmed that there is an “inclination” within the NHS to share information with the police and make a referral in order to avoid risks. However, they also acknowledged that Prevent is an essentially “grey area” in that “what we now get involved with is ‘if there’s potential’ – and this is where it’s really grey with Prevent” (CCG Prevent Lead B, 2017). This is particularly worrying as doubt is actively discouraged in the process: as a CCG safeguarding expert noted, what is looked for under Prevent is “a little bit of concern” about someone’s behaviour, which will be triaged by the safeguarding team – so the one who makes the referral doesn’t have to “own” it (CCG Safeguarding Expert C, 2017). As another CCG Prevent Lead admitted, “I’m not sure that [a referral] will help some people. I think it runs contrary to safeguarding under the Care Act in terms of how that works” (CCG Prevent Lead B, 2017).

Concerns about the potential inaccuracy and stigmatisation involved in this haphazard reporting structure are assuaged through policy discussion of the “pre-criminal space”. Prevent documentation, as well as interviewee statements, repeatedly emphasised that referees could come to little harm from the referral process – given that they would not obtain a criminal record or penalty. We also discovered that people referred to Prevent are often unaware that such a referral has been made. Their consent only needs to be obtained if the process reaches the stage where the Local Authority consider involving Channel. While policy documents encourage social care sectors to obtain consent when making Prevent referrals, they are not obliged to do so (NHS England 2017: 15–16). And, given the controversial and sensitive nature of a Prevent-referral conversation, our interviewees consistently left us with the impression that consent is usually sidelined (unlike in normal safeguarding procedures) or is fudged as a vague reference to “concerns” and obtaining unspecified “support” for the patient (CCG Safeguarding Expert C, 2017).

One particular Prevent trainer was particularly explicit about the contradiction they saw between the Prevent brief and normal safeguarding procedures of obtaining consent: “You’re supposed to ask their permission […] I don’t know whether anybody does. But you’re supposed to ask for their consent. 'I think you're being radicalised, I think you want to blow us up, would you mind if I referred you?' ” (Safeguarding Expert A, 2017).

But, regardless of not receiving a criminal record or even not knowing one has been referred, being put through a referral process could be highly stigmatising for individuals and breaks the trust established between health care and local communities. Several expert practitioners noted that “I think it’s very difficult to come back from a Prevent referral” (Safeguarding Expert A, 2017), and “that person’s life can be blighted [by a Prevent referral] in all sorts of ways. Professionally, personally, if you’re a child at school and nowadays, everyone knows you’ve been referred […] That label will stay with you, well after the investigation and possible trial” (Consultant Psychiatrist B and GP 2, 2017). The referral process was also thought to have broader negative effects upon the relationship between health care professionals and their local communities:

If that leaks out that you did an inappropriate Prevent referral, you break down your relationship with that community, your patients, that patient’s family. They’ll talk to other patients. She did the referral to Prevent. She thought I was a terrorist or whatever. It would be a total disaster, where you’ve worked so hard to create a safe space for these people to come and talk about some really sensitive issues. You have to think before you do that. (Consultant Psychiatrist B and GP 2, 2017)

In essence, safeguarding experts are aware of the imperfect fit between the Prevent Duty and safeguarding protocols. However, they mitigated those discrepancies by emphasising how their everyday (non-Prevent) safeguarding practice also sometimes exceeds the bounds of the Care Act, and by renegotiating aspects of the Duty. During our interviews, it became clear that some safeguarding experts assuaged their concerns about being implicated in predictive detection, by reframing the Prevent Duty as a way to reallocate resources to those deprived by the economic context of austerity. We now turn away from expert testimony to explore the everyday experiences of NHS staff with Prevent – specifically the training provided to NHS staff on Prevent and their interpretations of it.

## Description of the content of WRAP training

One of the many tasks of NHS safeguarding teams is to deliver WRAP training to Trust/CCG staff. The “WRAP” training lasts 1‒2 hours and involves the screening of clips from the Home Office provided DVD. Since 2016, WRAP trainers need not attend a “train the trainer” workshop; they are qualified by simply having previously attended a WRAP session and by achieving their line manager’s approval for taking on the role. In an NHS England National Prevent Update, cascaded to all safeguarding teams and prevent leads in the NHS in August 2016, staff were informed that

**Figure:**
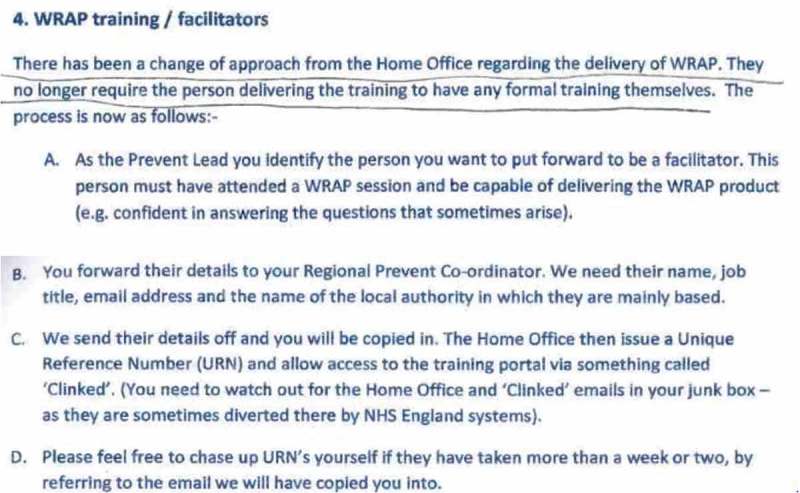

(NHS England 2016: 2–3)

No specific expertise is required to deliver Prevent training, because the role involves reading from the Home Office approve script for the session and interspersing the monologue with clips from the DVD. But in the course of our interviews with Prevent leads across the Midlands, it became clear that no one found it possible or effective to stick to the script. Some trainers reported that they use jokes about the training to lighten the atmosphere, whereas others integrated discussion of current events into the session.

The “Facilitators Workbook” provided by the Home Office suggests that trainers tailor their session to the level required to “achieve buy-in” from the audience. For management audiences, the Home Office recommend that this might only involve playing the “what is Prevent” clip alongside one of the case studies contained on the DVD. However, the workbook then states that there is a minimum content threshold for the session to be considered a WRAP. The relationship between full WRAP, and the recommended session for management audiences, remains unclear:

**Figure:**
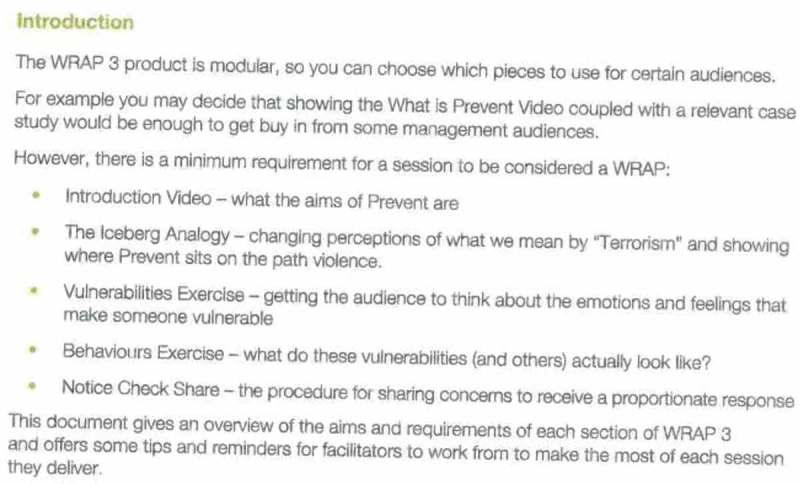

(Home Office undated: 2)

After the introduction of Prevent, the Workbook then instructs the trainer to proceed by choosing one of the case studies included on the DVD: either “Al Qaeda”-related radicalisation, or “Far Right”. Emphasis is placed on choosing the clip which “best suits your audience”, and ensuring that the second clip used later reflects a different ideological standpoint. Interestingly, the Facilitator’s Workbook then explicitly scripts the trainer in a fake “trust-building admission”, designed to win over their audience. The trainer is advised to reveal that they once found it hard to accept Prevent as a form of safeguarding, and how they overcame that perspective by “recognising that their conception of terrorism was too narrow”. This, the Workbook advises, will build trust that can later be exploited by “educating the audience” with the iceberg metaphor of terrorism and enable you to “assert your authority for the rest of the session”:

**Figure:**
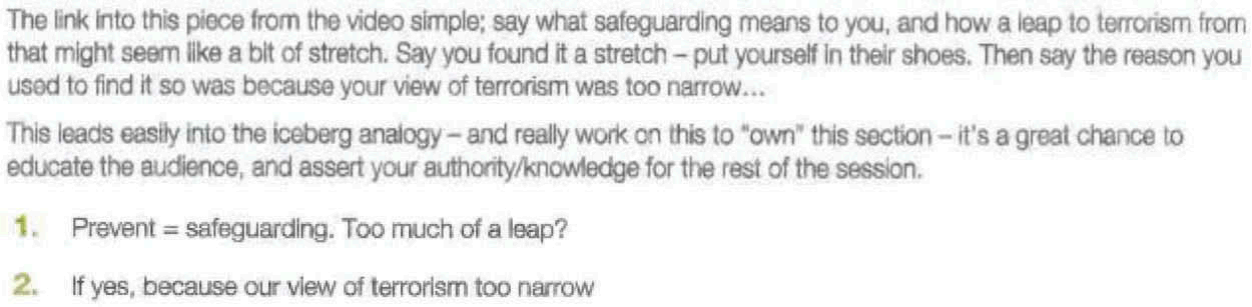

(Home Office undated: 4).

The Facilitator’s Workbook clearly shows that the training package aims to deflect criticism that Prevent does not fit within NHS safeguarding protocols. The aim of the training is not to engage open discussion but to embed the duty to report radicalisation concerns to one’s line manager or safeguarding team.

Similarly, the internal narrative of the Facilitator’s Workbook instructs trainers to control the discussion of case studies from the DVD. Rather than allowing flippant suggestions of vulnerability indicators, or “shouting out”, the trainer is repeatedly instructed to focus the discussion on emotional states that might lead to radicalisation:

**Figure:**
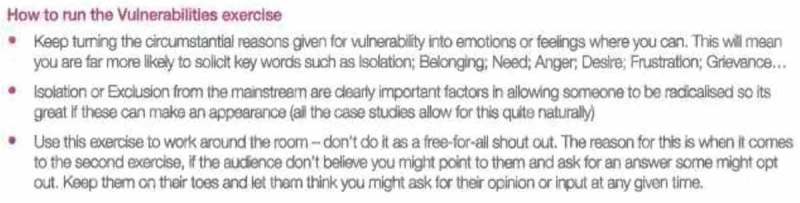

(Home Office undated: 6)

Through the policing of discussion possibilities, and the threat of being put on the spot by the trainer, the audience are drilled to accept vague emotional and social states (family upheaval; low self-esteem; social exclusion) as precipitating factors in radicalisation. NHS staff are never trained in specific behavioural signs that indicate radicalisation, but rather instructed to adopt an attitude of suspicion towards emotional states.

Given the multiple contexts in which family upheaval, low self-esteem and social exclusion are manifest, the indicators of radicalisation are presented in very broad terms within WRAP. So what scenarios do NHS professionals think they are supposed to report? The second stage of our study involved testing NHS staff attitudes towards the Prevent Training through a questionnaire, and asking what scenarios they consider to be reportable as a Prevent query.

## Everyday NHS staff attitudes to the prevent duty and WRAP training

Our questionnaire was developed to capture data around each participant’s pay grade, the NHS Trust/CCG for whom they work, their confidence in the WRAP training, the behaviours which they would consider reporting as a Prevent query and their perceptions of the social functions of the Prevent Duty (safeguarding or surveillance). The questionnaire was hosted on a University of Warwick server, gained ethical clearance from the University’s Biomedical Sciences Research Ethics Committee, and was distributed to the workforces of each trust and CCG participating in the study by their Head of Safeguarding. In total, 329 NHS staff completed the questionnaire.

The uptake between participating Trusts and CCGs was imbalanced: 76% of responses were drawn from a mental health trust in a non-priority Prevent area of the Midlands; 15% came from an Acute Trust in a non-priority area; 3% came from CCG staff in a non-priority area and the remaining respondents either did not specify their employer or were employed in various other trusts across the UK. These “others” discovered the survey through publicisation by the MEND network (Muslim Engagement and Development). We did attempt to obtain research access to Trusts and CCGs in Prevent priority areas but we were not successful.

**Figure 1.**
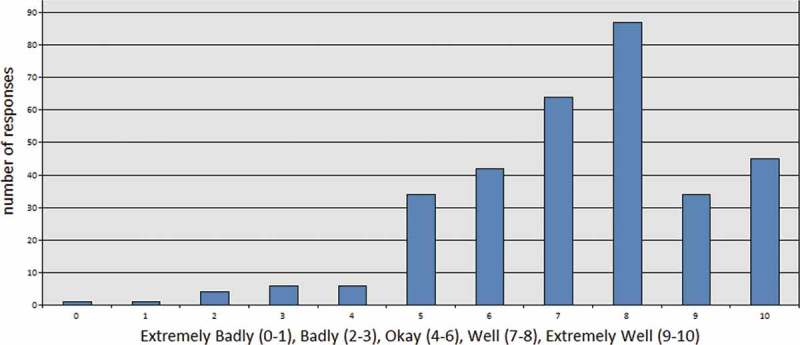
Grade how well prevent training explained the signs of radicalisation.

After obtaining contextual details about the participant’s pay grade (to establish the degree of seniority they possess) and previous safeguarding training received, we asked respondents to grade how well WRAP (or other forms of Prevent training) explained the signs of radicalisation. We were interested to know how NHS staff felt about the training, given that our content analysis of WRAP demonstrated significant vagueness around the communication of radicalisation signs. Interestingly, 71% of respondents graded the training positively (marking it as, or above, “7 out of 10”) in this regard (Figure 1).

**Figure 2.**
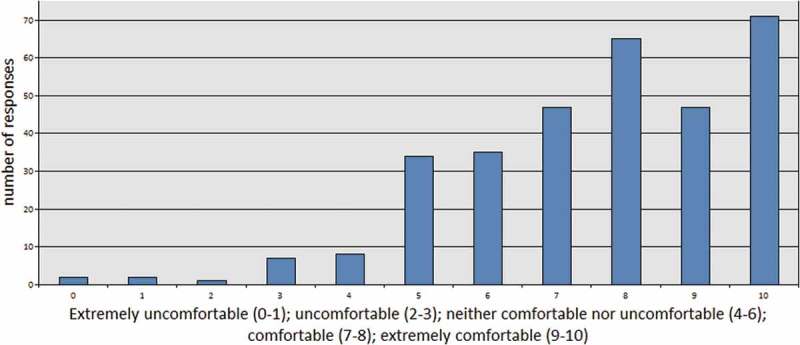
Grade how comfortable you were with the material presented during prevent training.

We interpret this positive reaction to the training’s communication of radicalisation indicators as also being an endorsement of radicalisation as the interruptible process which leads to terrorism. If a respondent was unconvinced by the radicalisation narrative put forth by government and media, then we argue that they would have marked the training as “bad” or “extremely bad” on this scale. Similarly, positive results were obtained when we asked participants to reflect on their comfort or discomfort with the material presented during their Prevent training. About 72% of respondents graded themselves as “comfortable” or “extremely comfortable” with the training materials (Figure 2).

**Figure 3.**
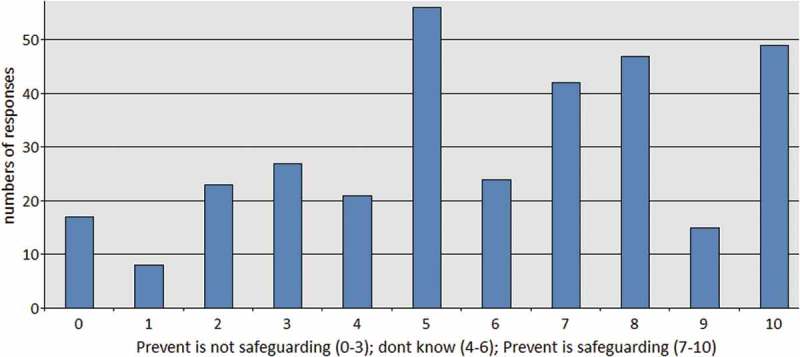
Prevent is just safeguarding. It is the same as safeguarding people from domestic abuse, financial abuse and sexual abuse.

At first glance, these findings tend to support the conclusions of a study which explores the roll-out of Prevent in education – specifically, that the majority of public sector respondents accept the core government narrative that Prevent should be understood as part of their safeguarding duties (Busher et al. 2017). However, when we directly asked health care professionals whether they understood the Prevent Duty to be a form of safeguarding, our results became far more ambiguous. Only 47% reported agreement with the statement that “Prevent is just safeguarding. It is the same as safeguarding people from domestic abuse, financial abuse and sexual abuse”. Slightly over 30% of people “didn’t know” whether Prevent fits the profile of safeguarding, whereas 22% disagreed with the statement (Figure 3).

This move towards ambiguity when directly questioned about Prevent’s status as safeguarding is very interesting. When compared to similar research in the education sector (Busher et al. 2017), it suggests that the context of safeguarding in health affects respondents' attitudes towards the Duty. In health care, unlike education, safeguarding is associated with protective intervention upon adults with “care and support needs”. Our results show that the majority of health care professionals (in our study) are accepting the Prevent Duty’s place in health and are comfortable with its requirements; however, this acceptance does not signify a similar acceptance that Prevent is safeguarding. Results for that question were significantly split, with less than 50% of respondents agreeing with the Department of Health’s framing of Prevent as safeguarding.

Our survey continued by exploring health care professionals’ attitudes towards various scenarios and whether they would constitute grounds for making a Prevent query. We developed these questions to probe deeper into the pedagogical effects of WRAP training. WRAP is quite vague on specific indicators of radicalisation and instead emphasises the wide-ranging emotional states which “make a person vulnerable” to extremism, like family upheaval, low self-esteem and social exclusion (Home Office undated: 6).

**Figure 4.**
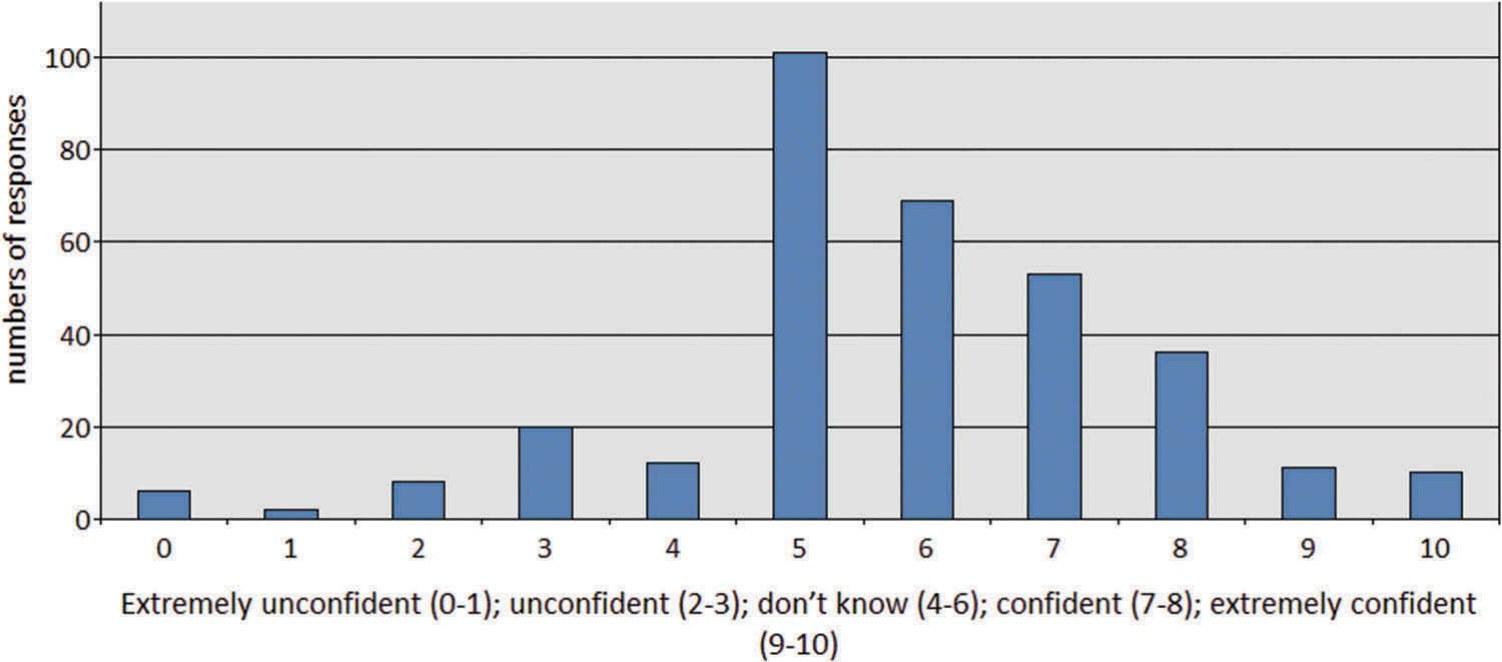
Grade how confident you feel to tell the difference between someone experiencing radicalisation and someone with an interest in middle eastern politics and wars.

One of the most significant findings was that health care professionals’ confidence in their ability to spot radicalisation fell away when we introduced mildly complex scenarios. For example, we asked respondents to grade their confidence in distinguishing radicalisation from someone’s interest in Middle Eastern politics and wars. Only one in three respondents reported having any degree of confidence that they could make the distinction; 56% of participants stated that they “didn’t know” if they could distinguish interest in Middle Eastern politics and wars from radicalisation and 11% were explicitly unconfident to tell the difference (Figure 4).

This represents significant ambiguity about the character of radicalisation and its relationship to someone’s general interest in current and/or political affairs. This finding was also apparent when we asked health care professionals’ to consider whether someone’s possession of radical philosophy necessitated making a Prevent Duty query. We deliberately did not qualify what we meant by “radical Islamic/anarchist philosophy” in the survey, to see if respondents queried the definition. It is notoriously difficult to classify philosophy as radical or non-radical. However, health care professionals felt extremely confident to understand the difference, without specific training on the matter. Only one respondent (from a sample of 329) left comments on the survey about potential difficulties in distinguishing radical from non-radical philosophy.

**Figure 5.**
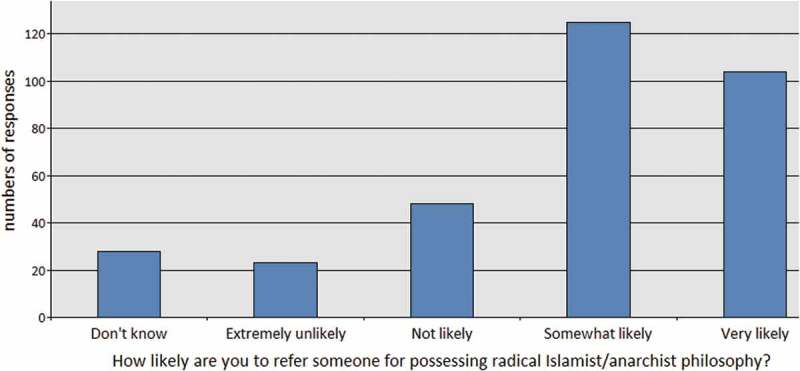
If a patient or staff member possessed books about radical Islamic philosophers or radical anarchism, how likely is it that you would make a safeguarding query?.

Leaving aside the unspecified nature of radical philosophy, 70% of respondents were “somewhat likely” or “very likely” to make a Prevent referral about someone on the basis of radical philosophy possession. Only 22% were “unlikely” or “extremely unlikely” to make such a referral. 8% said that they “didn’t know” if they would make such a referral (Figure 5).

We conclude from this that health care professionals feel inappropriately confident to judge whether philosophical books are radical, and that they are worryingly inclined to suspect radicalisation solely from the possession of radical philosophy. Academic research suggests that the philosophically and religiously literate are actually less susceptible to radicalisation (Coolsaet 2015; Roy 2011), and WRAP training makes no mention of philosophy books as an indicator of concern, so we believe that respondents are drawing their attitudes from popular culture rather than official training or academic research.

**Figure 6.**
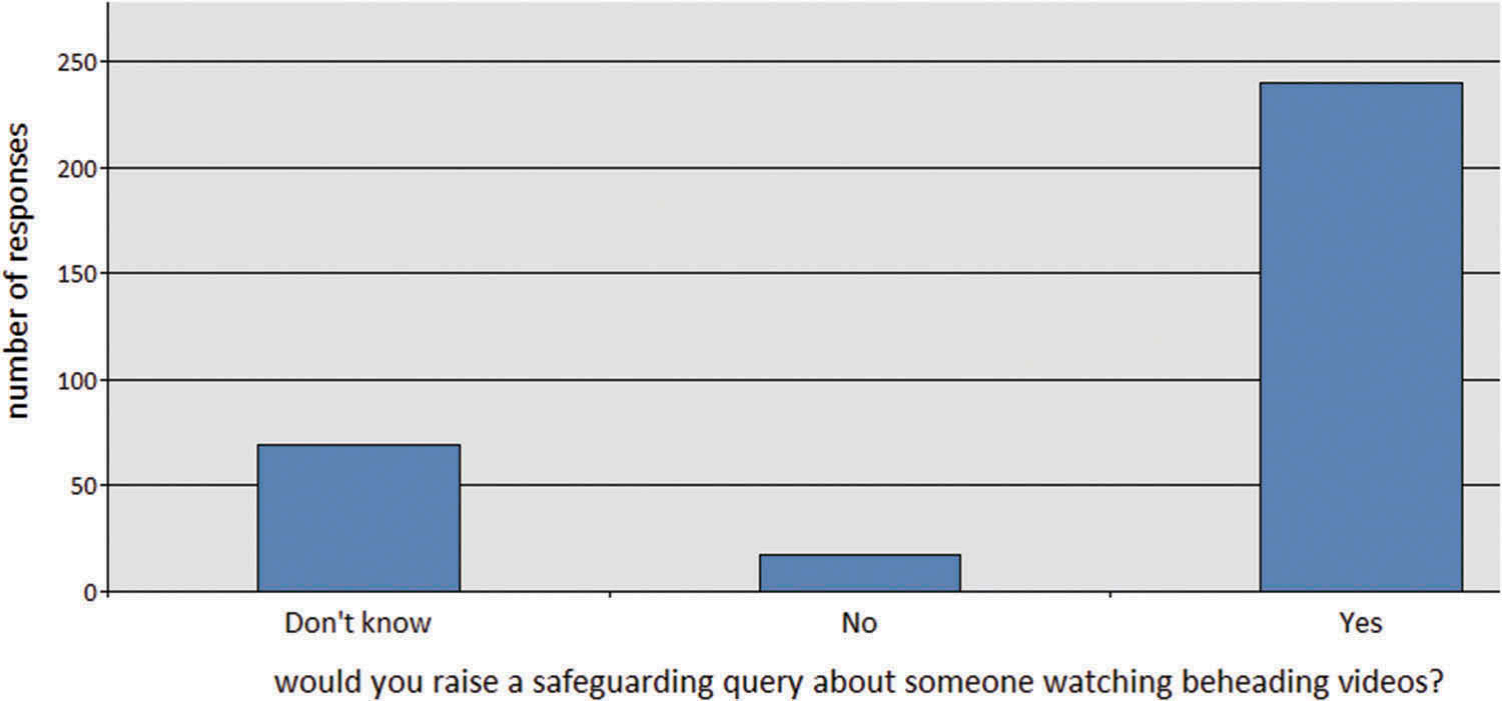
If you saw a patient or staff member watching video clips of beheadings, would you make a safeguarding query?.

Our suspicion that respondents are drawing their understanding of radicalisation from popular culture is further confirmed by their association of beheading videos with signs of concern. Beheading videos are not mentioned in Prevent training, so the association of viewing such videos with radicalisation comes from popular media. When asked if they would make a Prevent query about someone who watched beheading videos, 74% of respondents said “yes” (without any reference to the “care and support needs” generally needed for a concern to become relevant to safeguarding procedures); 21% “didn’t know”; and only 5% said they wouldn’t make a Prevent referral on this basis (Figure 6).

**Figure 7.**
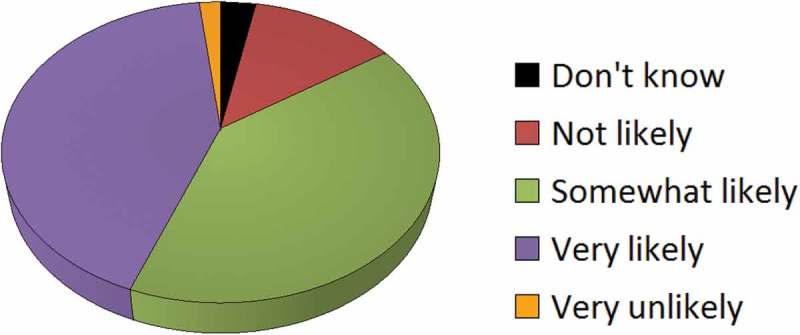
If a patient or staff member made hateful statements against an ethnicity, sexuality, or another minority group, how likely is it that you would make a safeguarding query?

Other criteria which health care staff strongly associated with radicalisation included hate speech about ethnicities, sexualities and other minority groups. An enormous 82% of respondents reported that they would be “very” or “somewhat” likely to make a Prevent query upon hearing such hate speech. Only 15% were “not likely” or “very unlikely” to make such a referral (Figure 7). While it is important to challenge hate speech in the workplace, the Prevent training does not specify that such illiberal opinion indicates radicalisation. However, upon the launch of the Prevent Duty, Education Secretary Nicky Morgan did identify vocal homophobia as a potential sign of radicalisation (BBC News 2015).^4^

However, interests in political events, possessing unpleasant opinions, or reading religious philosophy are not necessarily indications of radicalisation. NHS England has itself emphasised this in the most recent policy guidance for implementing the Prevent Duty:

Professionals should also have due regard to the Public Sector Equality Duty and be sensitive in their considerations. Outward expressions of faith or an interest in global or political events, or opinions that may seem unpleasant, in the absence of any other indicator of vulnerability or risk are not reason to make a referral to Channel. (NHS England 2017: 14)

And yet, our study indicates that NHS health care professionals in the Midlands are inclined to draw such conclusions and to potentially make Prevent referrals on these grounds.

## Conclusion

In the era of “after, after 9/11” in the UK, terrorism prevention has come to be framed as the extension of safeguarding. This framing of peer-to-peer counterterrorism surveillance as a banal measure of care has ensured the successful rollout of the Prevent Duty throughout the public sector. However, upon examination, there is an imperfect fit between Prevent and safeguarding in the health care sector (where Prevent is applied to people with formal care and support needs). We have raised concerns that the Prevent Duty deemphasises the formal care and support needs which legitimate protective intervention in an adult’s life, replacing them with vague (and circular) notions of vulnerability to extremist rhetoric. We also showed the various strategies health care professionals deploy to negotiate the tensions of Prevent in the NHS, including the highlighting of “grey areas” in normal safeguarding practice and the reframing of Prevent pathways as a way to redistribute care resources to those excluded by society.

Outside the realm of formal definitions and their interpretation by safeguarding experts, we also discussed the everyday experience of NHS staff with Prevent. After describing the content and pedagogy of the WRAP training product, we analysed the attitudes of 329 NHS staff to the Prevent Duty and their conceptions of radicalisation. Here, we found that the majority of those surveyed approved of the Duty, but were somewhat unconvinced of its status as genuine safeguarding. More concerning were the prominent trends within survey data which showed how staff associate radicalisation with philosophy possession and with hate speech. Illiberal attitudes and beliefs are being associated with radicalisation. Finally, we expressed grave concerns that only one in three respondents considered themselves confident to tell the difference between radicalisation and an interest in Middle Eastern wars and politics. Our survey results raise concerns that WRAP training generates a significant number of inappropriate referrals, some of which are removed from the system by the local safeguarding team, whereas others are deemed “misguided” by the Police Prevent Lead or Local Authority.

No figures exist for referrals which are deemed irrelevant or misguided by safeguarding teams or police. These fail to reach the Prevent Case Management system. But the figures for those referrals which do reach the Prevent Case Management system demonstrate a remarkably high attrition rate. Only 5% of formal Prevent referrals in 2016 and 2017 were allocated a deradicalisation mentor (Home Office 2017, 2018). Instead, the figures for 2017 show that 36% of formal referrals were abandoned,^5^ and 45% were given support from housing, education or health services. The remaining 19% were discussed at Channel Panel, of which a minority (5% of all formal referrals) received mentoring (Home Office 2018).

Before the statutory duty was introduced in 2015, this figure for mentoring (and thus the formal accuracy of Prevent referrals) was much higher. About 20% of referrals went on to receive mentoring support from Channel. But since the introduction of the Prevent Duty, the total number of referrals has jumped from 500–1000 per year to 7631 in 2015/16 (Home Office 2017). As Thomas Martin argues, this enormous increase in referral numbers – and the drop to 5% receiving Channel mentoring – reflects the responsibilisation of the public sector for counter-radicalisation and the prospect of censure for non-compliance (Martin 2018). Legal obligation has increased the numbers of referrals but not their quality.

This remarkably high failure rate of the Prevent Duty to identify subjects who require deradicalisation mentoring also speaks to the impact of the safeguarding framing and economic austerity. The provision of normal safeguarding support (housing, mental health care, education) to 45% of Prevent referrals distracts attention from the small number of people deemed to actually require deradicalisation intervention. But why could not these outcomes be obtained through normal social service provision, rather than a counterterrorism pathway? The context of economic austerity in the United Kingdom has diminished the capacities of the public sector to respond to social needs, and we find that the Prevent Strategy is gradually filling the gap – while simultaneously enabling surveillance (Heath-Kelly 2017a).

## Supplementary Material

Supplemental Material

## Notes

1. Clinical Commissioning Groups (CCGs) are NHS organisations set up after the Health and Social Care Act of 2012 to organise the delivery of healthcare services. Each CCG includes all of the General Practitioner groups in its geographical area, so that GPs and clinicians can influence commissioning decisions for their region.

2. The Netherlands’ “Integrated Approach to Combatting Jihadism” [Actieprogramma Integrale Aanpak Jihadisme Overzicht maatregelen en acties] policy of 2014 outlines the potential for youth workers and educators to prevent radicalisation: https://www.tweedekamer.nl/kamerstukken/detail?did=2014D2934&id=2014D29343 (last accessed 3 November 2017).

3. The Midlands has a population of over 10 million people and is constituted by the counties of Derbyshire, Herefordshire, Leicestershire, Lincolnshire, Northamptonshire, Nottinghamshire, Rutland, Shropshire, Staffordshire, Warwickshire and Worcestershire. The biggest cities in the Midlands are Birmingham, Coventry, Leicester, Nottingham and Stoke-on-Trent.

4. We are grateful to a reviewer for alerting us to this.

5. No figures exist for informal referrals which are removed from the pipeline by safeguarding teams or the initial police check. The real attribution rate for Prevent referrals is thus much higher.

## Interviews

Interview with CCG Prevent Lead B; 11 November 2017.

Interview with CCG Safeguarding Expert C; 1 October 2017.

Interview with Consultant Psychiatrist B and GP 2; 19 November 2017.

Interview with Safeguarding Expert A; 20 March 2017.

Interview with Safeguarding Expert P; 14 October 2017.

Interview with Safeguarding Experts F & J; 21 October 2017.
