# Supplementary material for: The banality of counterterrorism “after, after 9/11”? Perspectives on the Prevent duty from the UK health care sector
Source: Crit Stud Terror. 2018 Jul 17;12(1):89–109. doi: 10.1080/17539153.2018.1494123 (PMC6474731; doi:10.1080/17539153.2018.1494123)
Supplement: Supplemental Material [file RTER_A_1494123_SM0962.pdf]

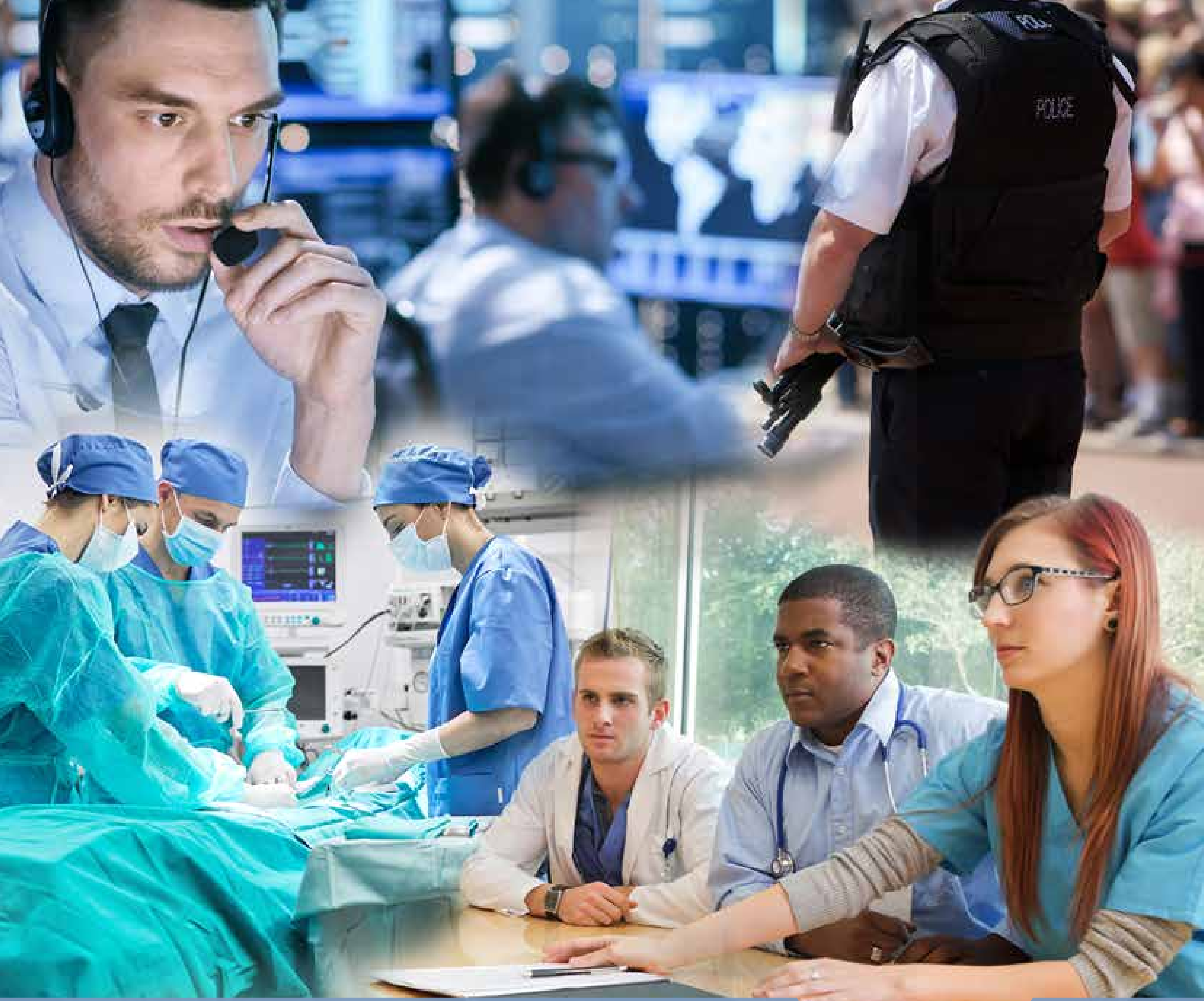

WARWICK

Counter-terrorism in the NHS

EVALUATING PREVENT DUTY SAFEGUARDING IN THE NHS

Authors:
Dr Charlotte Heath-Kelly, Dr Erzsébet Strausz

Executive Summary

Project Findings:

- The positioning of the Prevent Duty as a safeguarding measure is ambiguous. Safeguarding professionals alerted us that they are **operating in a 'grey area' with Prevent**, and that **significant differences exist between Prevent Duty safeguarding and normal safeguarding.**

- Our survey revealed that NHS staff are comfortable with the Prevent training provided and feel confident to detect radicalisation.

- However, our survey also revealed that NHS staff strongly identified hate speech, the possession of radical Islamic/Anarchist philosophy, and anger at foreign policy as indicators of radicalisation. **Prevent training modules do not identify these factors.** Staff would make Prevent queries to the safeguarding team if they noted these behaviours, which could be an inappropriate use of the safeguarding team's time.

- Our survey revealed that staff attitudes were polarised regarding whether Prevent is a form of safeguarding. **Only 47% of respondents agree that Prevent is a genuine safeguarding procedure, and 48% agree that Prevent belongs in healthcare.**

- NHS staff have very mixed opinions about whether Prevent is a form of surveillance, with **only 1 in 3 respondents confident that it is not surveillance.** However this did not affect a high level of confidence in individual Trusts/CCG's to make sensible and appropriate decisions about Prevent referrals.

- 4 Mental Health Trusts (of 54 in England) currently include radicalisation criteria in their Comprehensive Risk Assessments for all service users.

Conclusions and Recommendations:

- **We will contact the Health Select Committee to advise them of the gap separating the Care Act 2014 and Prevent Duty safeguarding.** We will raise the comments of safeguarding experts with them, regarding the expectation that they work in a legal grey area between the provisions of the Care Act and the Counterterrorism and Security Act.

- The line between mental illness and radicalisation is becoming increasingly blurred by initiatives like 'Prevent in Place', radicalisation screening practices in individual Mental Health Trusts, and the 2017 Prevent Guidance for Mental Health Trusts. **The Prevent Duty should not act as a fast-track pathway to mental health care, or other social services, as this creates incentives for well-meaning practitioners to misuse Prevent to access services.**

- Similarly, **mental health trusts should not integrate radicalisation criteria into Comprehensive Risk Assessments performed on all service users.** The Royal College of Psychiatrists Position Statement PS04/16 highlights concerns about the evidence base underwriting links between mental ill health and the likelihood of committing a terrorist act. Applying radicalisation screening to all service users risks inappropriately stigmatising the mentally ill.

- When making a referral to the safeguarding team, **NHS staff respond to the call for 'intuitive reporting' in WRAP by drawing from popular culture to understand radicalisation.** At the time the study was completed, images of ISIS flags and beheadings were prominent in the minds of participants. Safeguarding teams should be aware that these images will disappear from the media upon the defeat of ISIS, with unclear consequences for future Prevent referrals.

- **WRAP trainers should directly instruct staff to beware of unconscious bias when making Prevent referrals.** Currently there is a risk that popular culture stereotypes will influence staff perception of radicalisation.

Contents

Methodology & NHS Participation .... Page 6-7

Introduction: What is Prevent and why is it in the NHS? 8-13

What is Safeguarding? 14-18

Safeguarding Against Radicalisation 19-23

Testimonies of Prevent Leads and Safeguarding Experts............ 23-29

Is Prevent a Form of Safeguarding? Governance Standards 30-38

EU level documentation............ 32-33

NHS Safeguarding Governance............ 33

Information Governance 34-35

Counter-Terrorism Communications 36-38

How Does Staff Training Frame Prevent as Safeguarding............ 39-42

Survey with NHS Staff on WRAP training and Radicalisation Signs 43-55

Staff perceptions of WRAP training and Prevent 44-50

What behaviours do staff understand as radicalisation indicators...... 44-48

Discussion of Survey Findings............ 51-55

Radicalisation Screening, Prevent and Mental Health Trusts 56-60

Part 1:

Methodology & NHS Participation

Part 1: Methodology & NHS Participation

Methodology

In 2017, Dr's Heath-Kelly and Strausz ran a Wellcome Trust funded research project in the Midlands region. The funding was a Seed Award in the Humanities and Social Sciences (funder's ref: 205365/Z/16/Z), awarded to investigate how NHS trusts and CCG's have performed counter-radicalisation safeguarding under the Prevent Duty.

The Seed Award funded a pilot study – a type of small, initial research project, intended to break new ground and lead to bigger grant applications in the future. As such, this is not a nationwide study. But it does bring the expertise of experienced scholars of terrorism to bear on Prevent Duty safeguarding in healthcare. The Primary Investigator has published a book based on her research interviews with thirty ex-militants from insurgent groups in Europe.

The rationale for the study was that Humanities and Social Science research had not engaged with the topic of Prevent Duty safeguarding in the NHS. Furthermore, the United Kingdom is the only country in the world which has integrated counter-radicalisation activities into healthcare. As such, the team were funded to investigate:

- The NHS structures through which Prevent Duty safeguarding is performed;
- The acceptance (or otherwise) of counter-radicalisation as a safeguarding issue in the NHS (judged through interviews with safeguarding experts and a survey of Midlands NHS staff);
- The success of Health WRAP and WRAP training in embedding knowledge of the Prevent Duty within Trust/CCG staff (judged through interviews with safeguarding experts; a survey with Midlands NHS staff; and content analysis of the WRAP DVD);
- How such training operates pedagogically;
- How vulnerability to radicalisation is understood in the healthcare sector.

Who participated in the study?

- **Participating Organisations**: We spoke to safeguarding teams working in a mixture of six NHS Trusts and Clinical Commissioning Groups (CCG's) in the Midlands Area. One third of the participating organisations are located in primarily urban locations; two thirds were based in less metropolitan locations. None were in Prevent priority areas. We attempted to gain research access in these areas but were refused.
- **Interviews:** We conducted seventeen expert interviews. The experts included NHS Prevent leads, NHS Heads of Safeguarding, medics publishing in medical journals on the Prevent Duty and professional practice, one Channel Panel member, two Prevent Leads in the Police, and a forensic lead at a Prevent Mental Health Hub. Each interviewee has had the chance to check the transcripts of their conversation and to make amendments, and has approved the transcript for academic use and quotation.

Survey of NHS Staff Attitudes towards Prevent Duty Safeguarding:

Beyond our expert interviews, we conducted a survey experiment to test NHS staff attitudes to Prevent Duty safeguarding. Our questionnaire captured data around each participant's pay grade, the NHS Trust/CCG for which they work, their confidence in the WRAP training, the behaviours which they would consider reporting as a Prevent query, and their perceptions of the social functions of the Prevent Duty (safeguarding or surveillance).

The questionnaire was hosted on a University of Warwick server, gained ethical clearance from the University's Biomedical Sciences Research Ethics Committee, and was distributed to the workforces of each Trust and CCG participating in the study by their Head of Safeguarding. Each participating Trust and CCG has since received a tailored report on the anonymised attitudes of their staff members, with general themes and patterns indicated.

Participants received a £20 voucher for a popular online retailer, in exchange for completing the study. This incentive encouraged busy NHS staff to complete the 36 question survey. In total, **329 NHS staff completed the questionnaire**.

The uptake between participating Trusts and CCG's was imbalanced. 76% of responses were drawn from a Trust in a non-priority Prevent area of the Midlands; 15% came from an Acute Trust in a non-priority area; 3% came from CCG staff in a non-priority area; and the remaining respondents either didn't specify their employer or were employed in various other trusts across the UK. These 'others' discovered the survey through publicisation by the MEND network (Muslim Engagement and Development). We speculate that the imbalance in responses across Trusts/CCG's was partially caused by the size of distribution lists in each participating organisation, and relative degrees of desire for the compensation voucher.

Part 2: **Introduction: What is Prevent and why is it in the NHS?**

Introduction:
What is Prevent and why is it in the NHS?

Governments have long shared the ambition to prevent terrorist attacks. In the 21st century, many European states now try to prevent terrorism by intervening in the lives of those thought to be vulnerable to radicalisation. Support and mentoring packages are provided to prison inmates and disenfranchised youth within residential communities. However this approach has been dramatically extended in the UK.

The United Kingdom is the only nation in the world to deliver counterterrorism within its education, healthcare and social care sectors as safeguarding.

Preventing Terrorism in the United Kingdom

In 2015, the Counterterrorism and Security Act passed into law. Section 26 of this Act is better known as the 'Prevent Duty'. The Prevent Duty placed a legal responsibility on schools, nurseries, universities, healthcare providers and the social care sector to have 'due regard to the need to prevent people from being drawn into terrorism.'

Employees are trained to spot the signs of radicalisation – usually by watching a Home Office produced DVD called 'Workshop to Raise Awareness of Prevent' (WRAP). They are then instructed to report any concerns about radicalisation to their line manager or safeguarding team, and structures have been set up to manage this process. The safeguarding team initially triage these referrals followed by the police prevent lead, who judges whether a referral needs to be discussed at the Local Authority Channel Panel.[1]

Despite a long British history of terrorism and counterterrorism, the Prevent Duty is historically unique.

Never before has the UK trained its educators, medics and social care professionals to detect those who might become involved in terrorism.

But there are international parallels for the society-wide reporting of views considered extreme and potentially dangerous. For example, countries within the former Soviet Union actively encouraged citizens to report others to the police during the twentieth century, should they suspect unorthodoxy.

However, denunciation in the former Soviet Union was a repressive tactic used to suppress the population and prevent the emergence of opposition to dictatorial rule. So the context is very distinct from the UK.

The UK has made the public sector responsible for reporting extremist views or conduct under the rubric of ***safeguarding***. The intention is to notice someone's proclivity for extremist ideology and conduct ***before*** they become involved in criminal activity, and to help them better access services they might need.

In Prevent Guidance produced by NHS England, this redirection is situated within the **'pre-criminal space'**.[2] This is not a recognised term in criminology, social science, or the healthcare professions. Yet it appears in NHS Prevent Training and Competencies documentation before being cascaded down into local Trust documentation.[3]

[1] *Interview with Prevent Police Lead, January 2018.*
[2] *NHS England (2015) Prevent Training and Competencies Framework (Leeds: Nursing Directorate).*
[3] *David Goldberg; Sushrut Jadhav & Tarek Younis (2017) 'Prevent: What is Pre-Criminal Space?', BJPsych Bulletin 41(4), pp.208-11.*

The conflict in Northern Ireland involved a prolonged insurgency against the British government by the Provisional IRA. The term 'radicalisation' was never applied to participation in the conflict. Military, policing and intelligence sectors were used against the insurgency – the education, healthcare and social care sectors were not trained to spot future insurgents.

The Prevent Duty is an attempt to balance the United Kingdom's acceptance of liberal principles with the need to prevent terrorist attacks.

The description of Prevent as operating in the 'pre-criminal space' tries to balance the requirements of liberal democracy with the Prevent Duty. Countries which endorse political liberalism accept the need to protect free speech and debate: one cannot be criminalised for one's political views, however abhorrent they might be. The Prevent Training and Competencies Framework asserts the duty of healthcare workers to report anyone adopting 'increasingly extreme political, social or religious views' so that they can **receive support**, rather than be criminalised.

The Prevent strategy is focused on providing support and re-direction to individuals at risk of, or in the process of being groomed /radicalised into terrorist activity before any crime is committed (NHS England 2015: 6)

Prevent does not **criminalise** a person for their beliefs, or their verbal support of terrorist movements. Instead it uses a **safeguarding** approach; a measure used by Local Authorities to intervene in vulnerable people's lives to protect them from physical, sexual or criminal abuse. Radicalisation is compared to a form of grooming, whereby an abuser exerts control over a victim. This frames Prevent as protection, rather than repression.

But the positioning of Prevent as safeguarding, and terrorist recruitment as grooming, is contested.

Esteemed researchers of political violence have critiqued the predictive modelling used within radicalisation theories, as well as the racialized application of Prevent Duty safeguarding to people of colour.[4] Other scholars of terrorism show that ideological grooming is not a robust way to understand recruitment to militant groups, and provide robust sociological models of the protest cycles and structural factors involved.[5] Other academic research emphasizes the causal link between foreign military interventions and suicide terrorism performed against the invading nation.[6] Finally empirical studies also demonstrate a disconnection between a person's beliefs and their militant activity, showing that people disengage from militant groups without giving up their beliefs.[7]

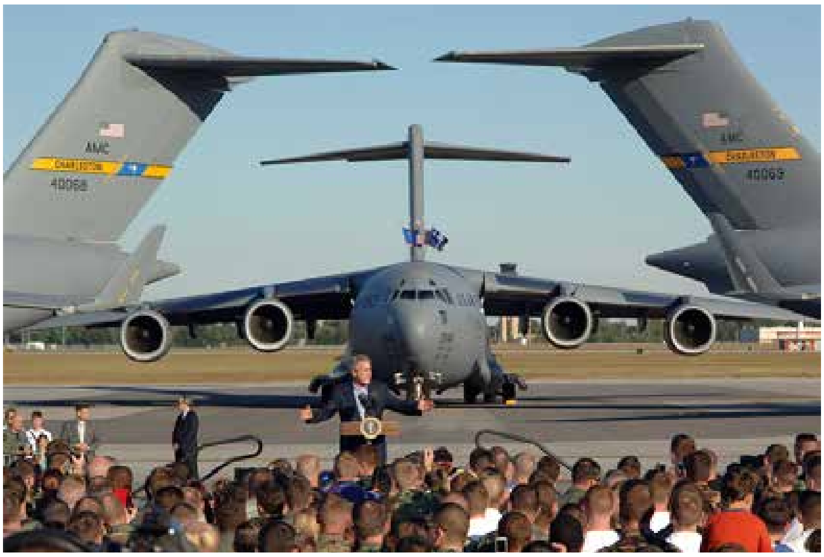

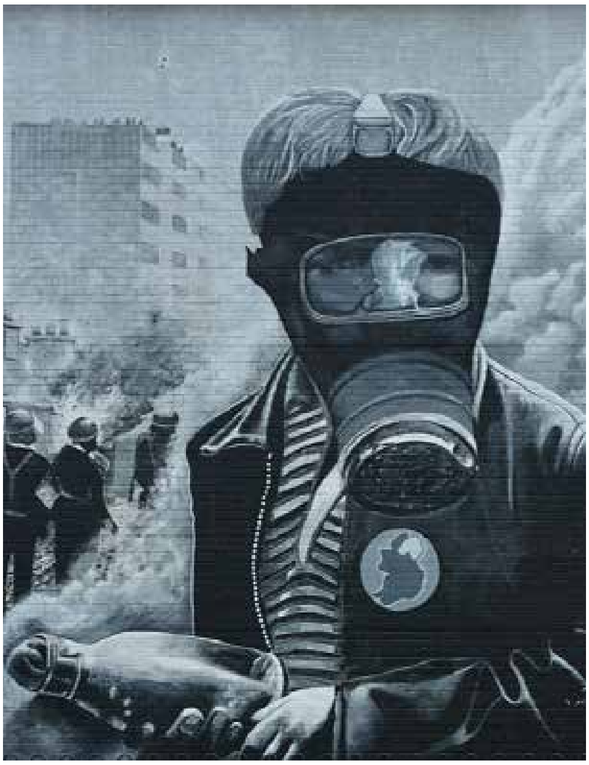

To conclude this introductory section, readers should note that ***radicalisation is understood as a metaphor by academics studying terrorism and political violence***, rather than as a process of grooming or recruitment. 'Radicalisation' is an analogy, or shorthand, used by policymakers when they want to discuss 'what happened before the bomb went off'.[8] Importantly, Mark Sedwick's discourse analysis of 'radicalisation' demonstrates that the term's usage rocketed in 2006 - but not in response to a scientific breakthrough in terrorism studies. Instead, the term's popularity is the result of policymakers and media requiring a term (a metaphor) to explain why British subjects would attack their own country.[9]

Reflecting academic research on political violence, the Home Affairs Select Committee has emphasized the wide variety of pathways into terrorism and condemned broad-brush governmental approaches to preventing radicalisation as counter-productive.

They stated:

> *"There is no evidence that shows a single path or one single event which draws a young person to the scourge of extremism: every case is different. Identifying people at risk of being radicalised and then attracted to extremist behaviour is very challenging. It also makes the task of countering extreme views complex and difficult. If the Government adopts a broad-brush approach, which fails to take account of the complexities, and of the gaps in existing knowledge and understanding of the factors contributing to radicalisation, that would be counter-productive and fuel the attraction of the extremist narrative rather than dampening it."*
> *Home Affairs Committee, 2016: 9.*

In this unique historical context, our research questions become:

If academia and Parliament recognize the extreme complexity of attempting to prevent people's involvement in terrorism, how are NHS staff tasked with preventing radicalization?

How are healthcare professionals trained to report the signs of radicalization, when there is no scientific consensus on relevant indicators?

[4] *Kundnani, Arun (2015) The Muslims are Coming! Islamophobia, Extremism and the Domestic War on Terror (London: Verso); Sageman, Marc (2016) Misunderstanding Terrorism (Philadelphia: University of Pennsylvania Press).*
[5] *Alimi, Eitan Y.; Chares Demetriou & Lorenzo Bosi (2015) The Dynamics of Radicalization: A Relational and Comparative Perspective (Oxford: Oxford University Press); Della Porta, Donatella (2013) Clandestine Political Violence (Cambridge: Cambridge University Press).*
[6] *Pape, Robert (2005) Dying to Win: The Strategic Logic of Suicide Terrorism (New York: Random House).*
[7] *Bjørgo, Tore & John Horgan (2009) (eds) Leaving Terrorism Behind: Individual and Collective Disengagement (Abingdon: Routledge).*
[8] *Neumann, Peter (2008) Perspectives on Radicalisation and Political Violence: papers from the first International Conference on Radicalisation and Political Violence, London, 17–18 January 2008 (London, International Centre for the Study of Radicalisation and Political Violence), p.4.*
[9] *Sedgwick, Mark (2010) 'The Concept of Radicalisation as a Source of Confusion', Terrorism and Political Violence 22(4), pp.479-94.*

Part 3:

What is safeguarding?

What is safeguarding?

The Department of Health presents the Prevent Duty as a patient safeguarding measure, which entails no extra responsibilities on behalf of clinical and non-clinical staff.[10] Prevent training and the processing of Prevent referrals sits with Trust and CCG safeguarding teams.

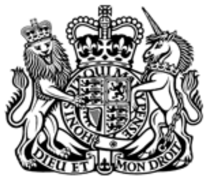

Children Act 1989

Safeguarding processes are designed to protect those with care and support needs (like learning disabilities, severe mental health conditions, and dementia) from abuse. They are a necessary societal protection for those with reduced individual capacity or agency.

Safeguarding has emerged organically from various pieces of legislation, professional standards of practice, and in response to high profile events. The 1989 Children Act formalized the UK's child protection procedures and put them on a statutory footing.[11] The societal duty to protect children from abuse, intervening to remove them from their family if necessary, is not particularly controversial. It is accepted that children require the protection of adults, given that they are more vulnerable and less able to protect themselves.

But the extension of these measures to adults has proven more difficult. There is no equivalent to the 'Children's Act' for adults and no codified description of adult protection. Extending state powers to intervene in the lives of adult citizens is a more complicated area.

Why is it more difficult to intervene in the lives of adults?

On the one hand, we know that some adults (especially those in care homes or with substance abuse problems) are preyed upon by criminals and abusers. However the desire to protect these adults has to be balanced against a societal commitment to the freedom of adults in general.

In a liberal democratic society it is customary to afford rights and liberties to adult citizens, and to restrain state intervention in their lives – for as long as criminality is not an issue.

Governing for intervention in the lives of non-criminal adults is a complicated area. Slowly, and via a piecemeal approach, the category of 'vulnerable adult' began to appear in policy guidance materials as a bridge. This bridging began when the Social Services Inspectorate forged a working definition of elder abuse in 1993, noting that such abuse could be physical, sexual, psychological or financial.[12] As Brammer and Biggs show, the guidance left some confusion as to the age at which one becomes 'an older person' – and therefore the legal boundary between domestic abuse and elder abuse.[13]

The threshold at which the state should intervene in an adult's life was more comprehensively considered in the Law Commission's 1995 report *Mental Incapacity*. Presciently, they expanded the terms of reference beyond incapacitated individuals to those who remained *vulnerable*.

---

[10] *Department of Health (2011) Building Partnerships, Staying Safe; The Health Sector Contribution to HM Government's Prevent Strategy: Guidance for Healthcare Workers (London: HM Government), p3.*
[11] *Mandelstam, Michael (2009) Safeguarding Vulnerable Adults and the Law (London: Jessica Kingsley).*
[12] *Social Services Inspectorate (1993) No Longer Afraid: The Safeguard of Older People in Domestic Settings.*
[13] *Alison Brammer & Simon Biggs (1998) 'Defining elder abuse', The Journal of Social Welfare & Family Law 20(3): 285-304.*

Vulnerability included:

> *"those over the age of 16 in need of community care services due to disability, age or illness, who are or may be unable to care for themselves or protect themselves from significant harm or exploitation"*[14]

These recommendations for a safeguarding framework were initially ignored. But in 2000 the Department of Health published the 'No Secrets' guidance document for the NHS. The terms 'adult protection' and 'protecting vulnerable adults' became prominent – even if they remained somewhat ill-defined and with sketchy boundaries.[15] Like the Law Commission report, the category of the 'vulnerable adult' borrowed from the definition used to assess eligibility for community care in the Community Care Act of 1990. A vulnerable adult is a person:

> *"Who is, or may be in need of, Community Care services by reason of mental or other disability, age or illness; and who is or may be unable to take care of his or herself, and who may be unable to protect his or herself against significant harm or exploitation".*[16]

This definition has established the formal criteria for safeguarding and protective interventions in citizen's lives. It was repeated in the 2014 Care Act. ***It forges a necessary social compromise between the general societal acceptance of adult agency (that adults are free to run their own lives, as they see fit, without state intervention) and the social requirement that the state intervene to protect some people from abuse.***

Adult Protection stretches beyond safeguarding

But as soon as that compromise between adult agency and protecting the vulnerable was struck in law, it was exceeded. A Social Care Institute for Excellence review of Governance in Safeguarding Adults Boards found that local authorities commonly exceed the threshold set out in 'No Secrets' (and the 2014 Care Act).

The research, commissioned by the Department of Health, showed that Safeguarding Adults' practices extend beyond populations with needs for community care services, and thus the formal definition of vulnerability.[17] Local Safeguarding Adults Boards' definitions of vulnerability varied significantly, from the strict formulation of care and support needs ***through to a broad interpretation of safeguarding work as a potentially limitless preventative endeavor***. Similarly, definitions of abuse varied from the formal definition of acts which violate human and civil rights, through to broader definitions of any act that could lead to harm.

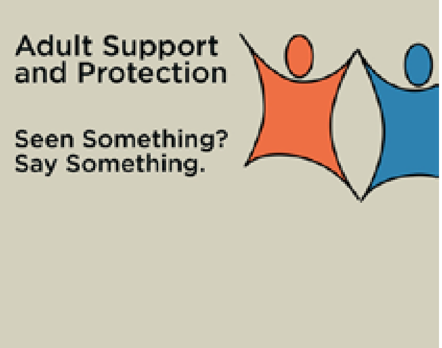

[14] *Law Commission (1995) Mental Incapacity (Law Com. No. 231), London: HMSO.*
[15] *Mandelstam, Michael (2009) Safeguarding Vulnerable Adults and the Law (London: Jessica Kingsley)*
[16] *Department of Health (2000) No Secrets: Guidance on Developing and Implementing Multi-Agency Policies and Procedures to Protect Vulnerable Adults from Abuse (London: HM Government), p.8.*
[17] *Braye, Suzy; David Orr & Michael Preston-Shoot (2011) The Governance of Adult Safeguarding: Findings from Research into Safeguarding Adults Boards (London: Social Care Institute for Excellence).*

Adult safeguarding is now recognized to operate:

- Beyond communities with Care and Support Needs
- In response to abuse and proactively to minimize anticipated harms.
- Against an increased number of abuses, including forced marriage, domestic violence interventions, and the provision of support to sex workers, drug addicts and those with mild/moderate learning difficulties (who can otherwise manage their own lives).[18]

[18] Braye, Suzy; David Orr & Michael Preston-Shoot (2011) The Governance of Adult Safeguarding: Findings from Research into Safeguarding Adults Boards (London: Social Care Institute for Excellence).

Part 4:

Safeguarding Against Radicalisation

Safeguarding Against Radicalisation

In 2011, the coalition government introduced radicalization as a form of abuse which safeguarding should prevent.

The Department of Health guidance for this new area of practice is split between emphasising the increased vulnerability of people with care and support needs to radicalisation, and the generalised vulnerability *of all patients, visitors and staff to radicalisation.*

At this point, the practice of Prevent Duty as safeguarding becomes conceptually unclear.

When radicalisation is presented as risk to people with care and support needs, radicalisation is framed as a type of abuse. But when radicalisation is presented as a condition which could affect anyone, it loses its association with care and support needs and is framed as a societal, and security, risk.

> *"Anybody, from any background can become radicalised. For the health sector, this may include colleagues or patients/visitors. This is a critical time to 'spot' people, as they are being radicalised."*
>
> *Royal United Hospitals Bath, available at http://www.ruh.nhs.uk/documents/PREVENT_extremism_and_radicalisation.pdf*

For example, the Department of Health initially situates Prevent in safeguarding by emphasising that 'people with mental health issues or learning difficulties may be more easily drawn into terrorism'.[19] Yet, later in the same document, 'changes in behaviour' become the criteria which should provoke a referral (regardless of care and support needs):

> *Through the course of their work, healthcare workers* ***may encounter changes in the behaviour of patients and/or colleagues*** *that are sufficient to cause them concern. A member of staff who has cause for concern will need to be able to raise this concern in the knowledge that it will be handled appropriately and that, where necessary, specialist advice and guidance can and will be obtained.*[20]

[19] *Department of Health (2011) Building Partnerships, Staying Safe; The Health Sector Contribution to HM Government's Prevent Strategy: Guidance for Healthcare Workers (London: HM Government), p.6.*
[20] *Ibid, p.16.*

Any person can be subject to a Prevent safeguarding referral, regardless of their formal capacity level. This drives a wedge between Prevent and safeguarding. PowerPoint slides from NHS England's Prevent update sessions highlight how vulnerability to radicalisation exceeds the Care and Support Needs threshold. As this example shows, 'anyone' could be vulnerable to radicalisation and all concerns should be reported:

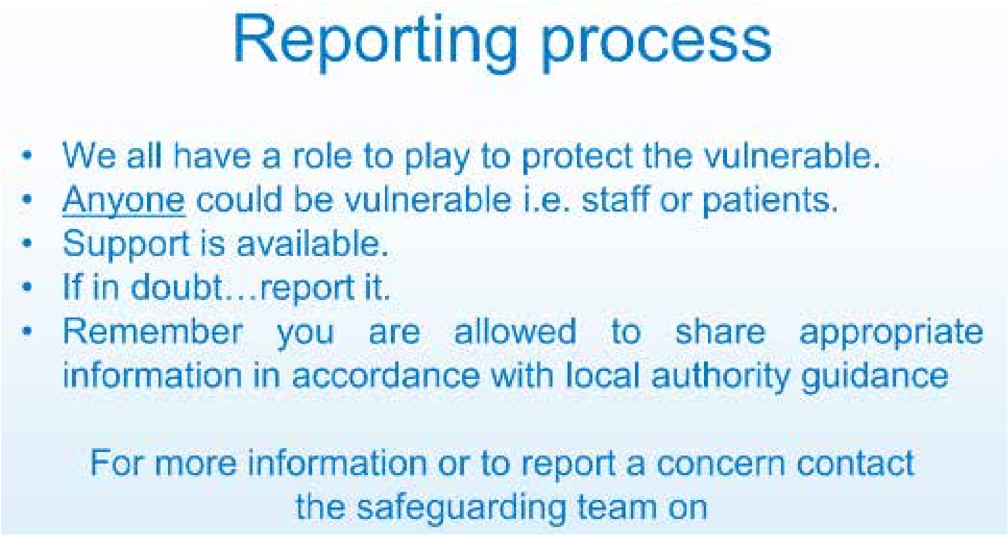

Source: Prevent Update for Internal Team Briefing, 2017

Furthermore, a Prevent Lead told us that NHS staff should apply the Prevent Duty to their colleagues, and people in their personal life, as well as NHS service users:

> *Some of these people, they might work in finance, but they might look over the shoulder of one of their colleagues and see them looking at some sort of thing on the internet or whatever and think 'hmmm'. But actually it's in your personal life - because it's happening all around us isn't it? So it's about that awareness bigger than just work […] When we speak to the staff here they think 'oh why do I need to do that?' And actually when they come to the session they understand, because actually they might be* ***living next door to somebody, they might see something going on. It's not just in your workplace. It's in the big wide world, you could be anywhere in the world couldn't you? Could be on holiday****, could be anywhere and you might see something.*[21]

[21] *Interview with CCG Safeguarding Lead A, 11th April 2017.*

Care and support needs are disassociated from the risk of radicalisation. But the Department of Health does provide some guidance on factors which put people at risk of exploitation by radicalisers. Worryingly, these racialise 'vulnerability' to extremism. The factors emphasize the migration status of an individual as a point of vulnerability to extremism, their 'traditional' family life (in a context where 'traditional' acts as a synonym for people of colour), and their 'religious/ cultural heritage'.

The first factor highlighted by the Department of Health as indicating radicalisation vulnerability is 'identity crisis'; but this is characterised as:

> *Adolescents/vulnerable adults who are exploring issues of identity can feel both distant from their parents/family* ***and cultural and religious heritage,*** *and uncomfortable with their place in society around them.*[22]

Identity crisis seems to be associated here with second or third generation immigrants, positioned between cultures.

The second factor in the Department of Health's list of radicalisation vulnerability factors is 'personal crisis'. This is characterised as the 'isolation of the vulnerable individual from the *traditional* certainties of family life'[23] [emphasis added]. Following immediately on from the first racialized indicator, this invocation of 'traditional family life' reads like a synonym for racialized groups in society.

The third factor in vulnerability relates to an individual's 'personal circumstances'. We all have 'personal circumstances', but these are instead characterised by the Department of Health in terms of cultural, religious and raced identities leading towards radicalisation vulnerability:

> *Personal Circumstances:* ***The experience of migration****, local tensions or events affecting families* ***in countries of origin may contribute to alienation from UK values*** *and a decision to cause harm to symbols of the community or state.*[24]

Finally, unemployment/underemployment, and criminality are listed (without racialized undertones) as factors which may make a person vulnerable to radicalisation.

Without a clinical evidence base or NICE guidance, these factors replace the formal care and support needs which are central to other forms of NHS safeguarding.[25] As a result, academic studies have questioned the transformation of safeguarding under the Prevent Duty, identifying a shift from safeguarding as a welfare-oriented practice to a security-oriented endeavour.[26]

This discomfort with this redirection of safeguarding was echoed in our interview with Consultant Psychiatrist B and GP 2:

---

[22] Department of Health (2011) Building Partnerships, Staying Safe; The Health Sector Contribution to HM Government's Prevent Strategy: Guidance for Healthcare Workers (London: HM Government), p.10.
[23] Ibid.
[24] Ibid.
[25] Care Act 2014, c.23, section 42 (London: Parliamentary Stationary Office).
[26] McKendrick, David & Jo Finch (2017) 'Downpressor Man: Securitisation, Safeguarding and Social Work', Critical and Radical Social Work 5(3), pp.287-300.
[27] Interview with Consultant Psychiatrist B & GP 2, 7th September 2017.

> *Anyone who knows anything about safeguarding can clearly see [Prevent] is not safeguarding, really. Because it's not transparent. There's no audit, there's no clinical governance. With sexual abuse, it's very clear what child sexual abuse is, and what you're protecting the person from. When you do safeguarding, the person sat in front of you is your main concern because you're trying to protect that person; whereas with [Prevent],* ***you're protecting the state from that person.***[27]

While it was rare for Safeguarding professionals to express this level of discomfort, they unanimously described how the Prevent Duty is not a perfect fit with safeguarding and the Care Act framework.

The Testimony of Safeguarding and Prevent Leads

Safeguarding experts commonly expressed support for Prevent as a form of protective intervention - but also emphasised that Prevent does not legitimately fit the safeguarding model. Interviewees often took both positions simultaneously. There was significant dissonance in many of the testimonies we collected for this project. We interpret this dissonance as the meeting point between individuals' commendable professional dedication to helping the vulnerable, and their awareness of the differences in protocol between Prevent Duty safeguarding and other safeguarding.

CCG Safeguarding Expert C moved between opposing understandings of Prevent quite frequently. Early in our interview, they affirmed that disenfranchisement and discrimination would make a person vulnerable to radicalisation and would legitimate safeguarding intervention:

> *"We need to see [radicalisation] as a non-criminal action rather than criminal action, and this is part of safeguarding vulnerable people […] Prevent, to me, is about finding people who are at the cusp in their lives of not having anything else, other than somebody saying 'why don't you do this?' and then being exposed to the sort of literature that gives you a purpose. And for a young Muslim child growing up, I suppose, who has a strong family bond and then suddenly is an isolated teenager, racially abused perhaps, can't find work, you're going to start connecting with things that you see and hear and read."*

The same interviewee later expressed **entirely opposite opinions** about the link between vulnerability and terrorism, and the nature of Prevent as safeguarding:

> *"I used to play in an Irish band and in the pub […] the jukebox was in Gaelic, you know, and the tin went around for the boys overseas; you know, in Ireland. And you just looked the other way, really. Those guys weren't vulnerable. Everybody that commits an act of terrorism doesn't need, necessarily, to be vulnerable. You have to have a belief system […] some people, particularly the organised groups, have committed acts of atrocities, haven't technically been vulnerable […]* ***The fact that Prevent sits under Safeguarding, I don't think it sits comfortably with Safeguarding."***

Similar dissonance appeared in other conversations with NHS professionals. Safeguarding Experts F & J gave testimony which demonstrated their confidence that radicalisation is a form of grooming abuse – but, without being prompted, also voiced a stern indictment of the distance separating Prevent Duty safeguarding from the Care Act. As experienced safeguarding trainers, they explained how resistance to Prevent amongst NHS staff was dropping and they less frequently deal with accusations of anti-Muslim bias during Prevent training sessions. They interpreted this as growing acceptance of radicalisation as a topic of relevance for NHS intervention, even if the provisions of the Care Act do not satisfactorily cover Prevent referrals:

> *It's a lot about people's vulnerabilities. People who will be targeted, people who will be drawn towards it [terrorism], are perhaps people who have got the vulnerabilities that we see in other sorts of safeguarding. Maybe, some mental health problems, maybe don't feel so loved and supported at home, feel a bit disenfranchised with society […] People are drawing them into it […]* ***The only way it doesn't fit with safeguarding, is really because other safeguarding, for adults, you would only really think about people with care and support needs.*** *So, that's how it sits under the Care Act. And, with Prevent, you haven't got that same thing, it's anybody. And,* ***the emphasis on normal safeguarding, as it were, is about making the person immediately safe. And, you know, you have to act quite quickly, perhaps. Slightly different with Prevent.***

Total Prevent referrals received by one trust's safeguarding team:

1. A nurse on an inpatient hospital ward reported an elderly gentleman, who had learning disabilities and lived alone. His behaviours were interpreted as indicating radicalisation. Safeguarding forwarded the case to Channel.

2. On a home visit to a family, a healthcare professional noticed a child sitting in front of an Arabic televised news channel. There were also Arabic reading materials lying around. The family were reported to social care as a potential case of radicalisation. The case did not reach Channel.

Finally, CCG Prevent Lead B gave a unique account of their engagement and simultaneous disengagement from the Prevent Duty. While they agreed with their regional prevent coordinator that complex needs increase vulnerability to becoming radicalised, they thought that the Prevent Duty was a response to inflated social anxiety about terrorism – rather than a genuine risk of widespread recruitment into terrorism. They concluded that they are very unconvinced about the effectiveness of Prevent, and emphasised that it runs counter to safeguarding in many ways:

People that end up either as lone wolf attackers or even get embroiled in radicalisation, a lot of them come from the same places as people who become mentally ill, who become murderers, who become drug addicts […]but I served in Northern Ireland, so you do pick up ways of thinking about some of this stuff. Rather than just saying: 'Well, we need to do it because it's dangerous out there and there's all these people around', ***which I don't necessarily believe for a second. It's more to do with lots of other things […] it's almost becoming a big, inflated anxiety in terms of what's going on*** *[…]If I got back to my computer and there's a big thing from the BBC and there's an email from NHS England and the Department of Health says: 'Right, we're going to stop this [Prevent] now'.* ***I'm really not sure what***

difference it would make [...] I'm not sure that that [Prevent] will help some people. I think it runs contrary to safeguarding under the Care Act in terms of how that works.

Prevent Referrals:

Safeguarding Expert A recounted 2 prevent referrals:

1. A Prevent referral where 'an Asian man' was reported to the safeguarding team for discussing his future trip to Saudi Arabia. This had been interpreted by another healthcare professional as a cause for concern, rather than a trip to participate in the Hajj pilgrimage.

2. An emergency department referred 'an Asian man' directly to the Police for arriving with burned hands, but not explaining how his hands became injured. This was interpreted as experimentation with bomb-making.

Resolving dissonance around Prevent and Safeguarding

Of course, not every safeguarding professional remained conflicted about the relationship between Prevent and the Care Act. In a minority of cases, interviewees found ways to overcome the professional ambiguity.

One Prevent Lead emphasised that normal safeguarding is legitimate but that the Prevent Duty is not safeguarding, it is an abusive form discriminatory surveillance. Concomitantly, a Head of Safeguarding from a different Trust took an alternate path to resolve the tension between safeguarding and Prevent. They emphasised that *all forms of safeguarding* (physical, financial, sexual) are imperfect and require professionals to operate in 'grey areas'. Prevent is therefore not exceptional in this regard.

Both resolutions of the ambiguity are worrying. To practice Prevent Duty safeguarding as an NHS expert, one must either consider the Prevent Duty to contradict with safeguarding, or one must hold the belief that all forms of safeguarding require teams to work in legal grey areas beyond the stipulations of the Care Act.

Safeguarding Expert A, who works as a Prevent Lead, espoused the former argument:

You could be doing us for discriminatory abuse when we make referrals. ***How we talk about vulnerability in terms of Prevent, is nothing like how we talk about vulnerability in terms of Safeguarding. Because, they've got to have care and support needs.*** *And with Prevent we're told it's about, you know, drug and alcohol abuse, and having issues with the system, and you have all these negativities that build up and stuff. And that's how the couch it in Prevent terms. It's a completely different thing.*

Prevent Referrals:

Safeguarding Expert Y recounted an example:

"I think back to a referral we made into Channel who was a doctor, who it was really hard to find any care and support needs for. And certainly, we weren't doing it under the safeguarding umbrella, but it was quite clear that the doctor posed a risk to the wider society."

Safeguarding Expert A emphasised that the responsibility placed on healthcare staff to notice signs of radicalisation was impractical. They explained that someone committed to terrorism would be smart enough to not reveal their intentions to NHS staff. Furthermore, Safeguarding Expert A wondered how a nurse or doctor was expected to notice signs of extremism over 'the ten minutes it takes to get your hand stitched up'. Any conversation that could potentially reveal such signs would likely be highly inappropriate in the context of healthcare.

Finally, Safeguarding Expert A expressed deep distress about the time constraints on their role. Prevent Leads are not given extra time to deliver Prevent training sessions, but must balance this against their existing safeguarding duties. **Safeguarding Expert A explained that they had been urgently dealing with finding housing for a victim of human trafficking, but had been abruptly removed from this duty and sent to deliver Prevent training at a different location.** Safeguarding Expert A perceived these duties to be of staggeringly different levels of importance and is upset that crucial safeguarding tasks are put in the same remit as Prevent Duty safeguarding.

Other professionals resolved the tension between the Care Act and the Prevent Duty in other ways. Safeguarding Expert Y is a Head of Safeguarding in an NHS Trust. Unlike Expert A, Expert

Y did not understand Prevent to involve much deviation from normal safeguarding practices. Instead Y took a very pragmatic perspective on the political factors which led Prevent to be integrated into the safeguarding brief:

My interpretation of what happened was that nobody would take this on; everybody said it doesn't fit into my agenda. And the then Chief Nurse said, 'it might not fit into anybody's agenda, but it's important. How can we align things so that it fits into somebody's portfolio?' And the view was, this is about protecting the vulnerable from exploitation, that's safeguarding, like it or not. ***We also have to acknowledge that not everybody fits comfortably under the Care Act part. […] it's been linked into safeguarding to sell it, to get somebody to own it.***

This reflective take on how the Department of Health, NHS and Home Office negotiated the placement of Prevent was accompanied by a similarly pragmatic perspective on the day-to-day fit with safeguarding. Expert Y recognised the imperfection which runs across all adult safeguarding. They told us that normal adult safeguarding often does not fit comfortably with the Care Act either,[28] leaving 'grey areas' to which safeguarding professionals must adapt their practice:

The Care Act was not helpful, there are a lot of people who we would like to support who are not encompassed under that heading, so we identify the issue and then have to find a creative way to find some support for them. If somebody has mental capacity and says, "I know my grandson is financially abusing me but I'm not giving you permission to tell the Police", my hands are tied unless I can find another elderly relative who doesn't have capacity who is possibly being preyed on by that grandchild, so it's very difficult. ***I don't have a problem with grey, it's my everyday bread and butter with [safeguarding] adults, with children it's so much more black and white*** *[…] So I'm comfortable with the fact that it doesn't fit completely into different boxes, it makes*

[28] *See also: Braye, Suzy; David Orr & Michael Preston-Shoot (2011) The Governance of Adult Safeguarding: Findings from Research into Safeguarding Adults Boards (London: Social Care Institute for Excellence).*

me think every time I'm choosing to share information on what grounds am I doing it? But those grounds might not be safeguarding, but so what; somebody's identified a problem, they've come to an expert and we've directed them.

The testimonies of Safeguarding Experts and Prevent Leads demonstrate a significant tension between the provisions of the Care Act, safeguarding adults protocols and the expectation that NHS staff make Prevent referrals. Professionals dealt with this in different ways; most lived with the dissonance and might not be aware that they made contradicting statements, whereas experts A and Y resolved the tension in very different ways. Expert A separated the Prevent Duty from normal, acceptable safeguarding. Expert Y viewed all adult safeguarding as often operating in a grey area, beyond the scope of the Care Act.

To further investigate this issue, we began to look at the situation of the Prevent Duty in NHS Governance.

Part 5:

Is Prevent a Form of Safeguarding? Governance Standards

Is Prevent a form of Safeguarding? EU level documentation

While the United Kingdom is, so far, the only nation to train healthcare professionals in the reporting of radicalisation concerns, there are ambitions to engage the health sector in counter-radicalisation across Europe. The 'Radicalisation Awareness Network' (RAN) is a European Commission Centre of Excellence, connecting policymakers and practitioners across Europe.

In January 2016, the Health and Social Care working group was set up within RAN, to compare and further the role of the health and social care sectors in radicalisation prevention across Europe.

Its work is divided between identifying and treating lone actors, as well as generating concrete proposals for multiagency structures which prevent radicalisation at the general level (i.e. across the population).[29] Meetings of the RAN Health and Social Care working group regularly attract policymakers as well as senior health and social care officials – including the Home Office Head of Communications Abu Ahmed, and NHS England's Director of Nursing NHS Hilary Garrett,[30] as well as Regional Prevent Coordinators and Prevent Leads from the NHS.[31]

In May of 2016, the Health and Social Care group produced a paper on how to set up a multiagency structure to prevent radicalisation. Importantly this paper advised European member states that the counter-radicalisation function of this multiagency structure should not be explicit or overt. **To avoid the perception that health and social care are involved in stigmatising people as radicals or extremists, the structure should focus on crime prevention in general:**

"Avoid stigmatising and labelling by setting up a more general structure: An overall multi-agency structure focused on different kinds of social issues which for example serves the more general aim of crime prevention and integrates the prevention of radicalisation dimension rather than making it the main objective of the structure, prevents stigmatising and labelling people as a radical person, violent extremist or even terrorist." [32]

[29] *RAN H&SC Identifying and treating lone actors, Zagreb 27-28 January 2016, available from https://ec.europa.eu/home-affairs/sites/homeaffairs/files/what-we-do/networks/radicalisation_awareness_network/about-ran/ran-h-and-sc/docs/ran_h-sc_identifying_and_treating_lone_actors_zagreb_27-28012016_en.pdf*
[30] *Ibid, p.10.*
[31] *RAN H&SC meeting on Multi- or cross-cultural approaches to preventing polarisation and radicalisation, Dublin 04-05 July 2017, p.9, available from https://ec.europa.eu/home-affairs/sites/homeaffairs/files/what-we-do/networks/radicalisation_awareness_network/about-ran/ran-h-and-sc/docs/ran_hsc_multi_cross_cultural_approaches_dublin_04-05_07_2017_en.pdf*
[32] *RAN H&SC Handbook on How to set up a multi-agency structure that includes the health and social care sectors? Copenhagen, 18-19 May 2016, p.3, available from https://ec.europa.eu/home-affairs/sites/homeaffairs/files/what-we-do/networks/radicalisation_awareness_network/ran-papers/docs/ex-post-paper-handbook-ran-hsc-18-19-may-2016-copenhagen-dk_en.pdf*
[33] *Ibid.*

Furthermore, **framing the multiagency structure as safeguarding was thought to be important for obtaining the cooperation of practitioners and receiving intelligence from them:**

"Building a more general structure around for example safeguarding children and vulnerable adults is also beneficial when it comes to wanting [sic] to receiving additional information from for example schools or youth workers." [33]

Here, the framing of counter-radicalisation as a safeguarding measure is described as a pragmatic measure to ensure successful information capture. It softens the appearance of counter-terrorism surveillance within the health sector and increases the level of acceptance which can be expected.

With the participation of senior Home Office, NHS executives and Regional Prevent Coordinators in the meetings of the working group, we can be assured of their input into this documentation. This evidence suggests that the framing of Prevent as a safeguarding measure is a pragmatic choice designed to maximise practitioner cooperation for information capture (surveillance) and to avoid public opposition.

Is Prevent a form of Safeguarding? NHS Safeguarding Governance

The researchers contacted NHS England with a Freedom of Information Request to clarify how NHS governance situates Prevent. We wanted to know how many Trusts have formally integrated Prevent into safeguarding, and how many treat it as a stand-alone policy (and not formally part of safeguarding).

The response confirmed a lot of what Safeguarding Experts told us about the problematic situation of Prevent within adult protection. By the second quarter of the year 2017-18, **only 27% of Trusts in Prevent priority areas of England had integrated Prevent into their Safeguarding policies** and 73% had stand-alone Prevent policies. In governance terms, the positioning of Prevent as a safeguarding measure is highly ambiguous:

Priority Trusts - Prevent Results Q2 2017-18

Q24 Prevent Policy - Stand-alone or integrated?

**Acute**	
Stand-alone policy	28
Integrated policy	14
**Specialist**	
Stand-alone policy	7
Integrated policy	5
**Mental Health**	
Stand-alone policy	21
Integrated policy	3
**Community**	
Stand-alone policy	7
Integrated policy	3
**Ambulance**	
Stand-alone policy	5
Integrated policy	2
**Care Trusts**	
Stand-alone policy	4
Integrated policy	0
**Overall**	
Stand-alone policy	72
Integrated policy	27
**Total**	**99**

Separately, we sent the same FOI request to all Mental Health Trusts in England (not just in Prevent priority areas) to compare the results. 54 Trusts responded. 14 of those trusts had integrated their Prevent policies into Safeguarding, whereas 40 have stand-alone Prevent policies.

26% of Mental Health Trusts in England treat Prevent as a Safeguarding policy in terms of governance. This result is only 1% less than NHS England's centralised figures for all trusts in priority Prevent Areas.

These low figures for integrated Prevent policies could be explained by the time it takes Governance Boards to amend Trust policies. But while the statutory Prevent Duty has been in force since 2015, work began to integrate Prevent into the NHS in 'priority areas' in 2011. **Six years is a considerable length of time – more than enough to make amendments to governance documentation – so the figures for Trusts in Prevent priority areas are surprising**. Prevent appears to be alienated from safeguarding in terms of governance, as well as professional practice. To further investigate the situation of Prevent in NHS safeguarding, we turned our attention to Information Governance policy.

Is Prevent Safeguarding? Information Governance Policy

NHS England policy shows us that safeguarding professionals and Information Governance Leads have raised concerns about the duty to refer adults without care and support needs, under the banner of Information Sharing.

In July 2017, the NHS England Prevent Team addressed staff concerns about breaching patient confidentiality when making Prevent referrals. The Practical Guidance document for Information Sharing and the Prevent/Channel Process states:

> *"1.3 The guidance has been developed* ***in response to concerns raised by health care practitioners*** *about information sharing for the purposes of Prevent and Channel particularly when:*
>
> *They are requested to share information without the individuals' prior consent or*
>
> ***The individual has not been explicitly identified as being at risk of harm, abuse or exploitation".***[34]

In normal safeguarding, the NHS would be expected to obtain the consent of the individual to share information, in order to protect them from abuse via multi-agency cooperation.

But **NHS procedures for information sharing vary between Prevent referrals and normal safeguarding**. Unlike normal safeguarding, it is rare for NHS personnel to obtain the consent of the person being referred to Prevent to share their information. Instead the referral is passed to the Police Prevent Lead who screens the referral to remove any cases where a counter-terrorism investigation is already taking place,[35] and checks the relevance of the case to Prevent.

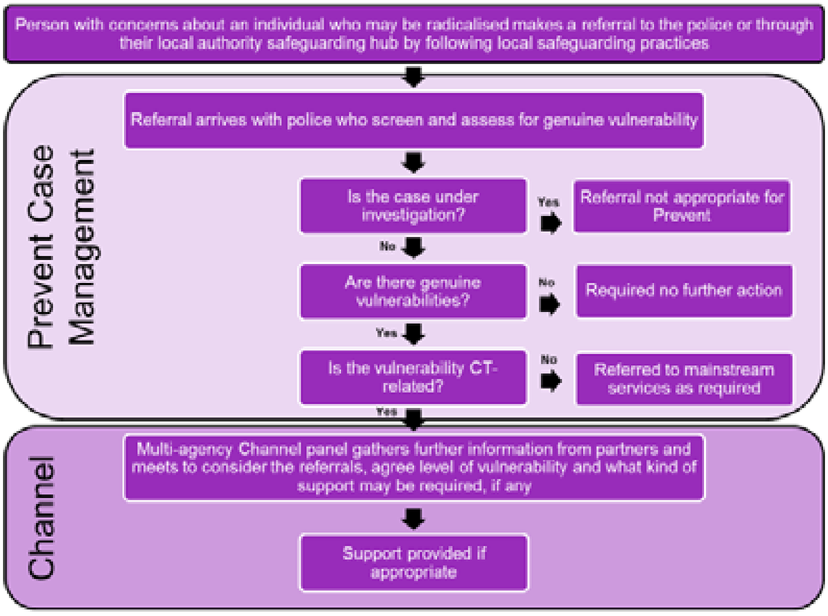

Source: Home Office (2017) Individuals Referred to and Supported Through the Prevent Programme, April 2015 to March 2016 (London: HM Government), p.6

[34] *NHS England Prevent Team (2017) Practical Guidance on the Sharing of Information and Information Governance for all NHS Organisations specifically for Prevent and the Channel Process (Leeds: Nursing Directorate), p.5.*

[35] *Home Office (2017) Individuals Referred to and Supported Through the Prevent Programme, April 2015 to March 2016 (London: HM Government), p.5-6.*

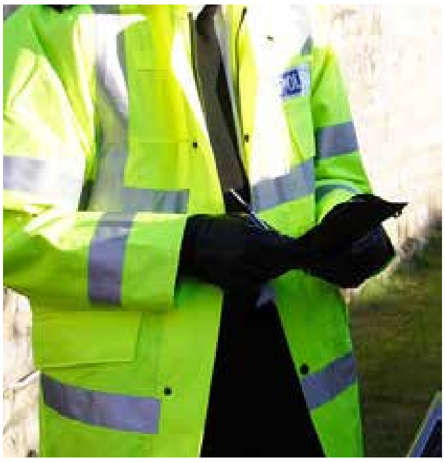

Our interview with a Police Prevent Lead revealed that they then perform a home visit to check the referral for 'the 3 M's' – misguided, malicious and misinformed referrals. If the concern is felt to be relevant to the Channel process, the Police officer then obtains the person's consent to share their information for the purposes of obtaining support. Consent is given by signing the Police Officer's notebook, or the form produced by some forces for this purpose.[36]

Unlike information sharing in normal safeguarding, the NHS rarely obtains consent. **Prevent referrals use the 'prevention of crime' exception (s.29) to the Data Protection Act to enable information sharing between the NHS and the Police.** As the NHS' Guidance notes on Information Sharing under Prevent state, 'Section 29 allows a Data Controller to proactively disclose information to the police, as long as the purpose is for the prevention/detection of crime, or the apprehending of offenders'.[37] This guidance on Information Sharing is intended to reassure concerned practitioners that they are acting within the law when they make Prevent referrals. However, **it highlights the profound distinction between the protection of vulnerable adults experiencing abuse and the prevention of crime**.

This Information Governance protocol marks a distinction between normal safeguarding and the Prevent Duty. It places the referral in the context of crime prevention, rather than adult protection.

Undertaking Prevent referrals under the crime prevention exemption of the Data Protection Act also involves tension with other NHS protocols. In 2012, the Department of Health published its response to the 'Liberating the NHS: No Decision About Me, Without Me' consultations. Greater patient involvement in decisions about their care is a central theme within the document. Beyond introducing a greater degree of choice in healthcare provider, the document emphasises the need for a culture change in the NHS to make patient involvement in care decisions routine.[38] And yet patients are not involved in decisions to refer them to Prevent as a safeguarding concern. They are excluded from decisions about their care by the NHS, until their case is verified by Police as relevant to Channel.

Not telling patients that a Prevent concern has been raised about them runs counter to the aims of 'No Decision about me, without me'.

---

[36] Interview with Police Prevent Lead, 12th January 2018. The Police Prevent lead also emphasised that it is not always the police who obtain consent, and gave examples of schoolteachers undertaking the conversation.
[37] NHS England Prevent Team (2017) Practical Guidance on the Sharing of Information and Information Governance for all NHS Organisations specifically for Prevent and the Channel Process (Leeds: Nursing Directorate), p.8.
[38] Department of Health (2012) Liberating the NHS: No Decision About Me, Without Me (London: HM Government).

Is Prevent Safeguarding? Counter-Terrorism Communications

The situation of Prevent as a safeguarding measure is further complicated by the counter-terrorism communications and briefings received by each safeguarding team. NHS safeguarding teams have been integrated within a Home Office structure which oversees their implementation of the Prevent Strategy. Each Prevent Priority Area in England has a designated Regional Prevent Coordinator (RPC) who is seconded from the Home Office to the NHS to support and embed the Prevent Duty.[39] **RPC's and the Home Office regularly cascade communications about counterterrorism matters to NHS safeguarding teams, blurring the line between security operations and safeguarding.**

For example, after the Manchester Arena bombing of May 2017 safeguarding teams were instructed to notice patients who presented themselves as having been at the Arena and report them immediately to West Yorkshire Police. Safeguarding teams were exposed to communications which blurred the provision of healthcare with the detection of crime witnesses and terrorists:

> Dear Colleagues
>
> The North East Counter Terrorism Unit (NECTU) are leading the Police investigation in to incident at the Manchester Arena on 22nd May 2017.
>
> They have requested that any patient or member of the public who identifies themselves as being involved in or affected by the incident be encouraged to call **101** and ask for **WEST YORKSHIRE POLICE.** (West Yorkshire Police are leading the coordination on behalf of NECTU).
>
> Q. What if the patient or the member of the public doesn't want to ring 101?
> *A. You can't make them call 101 but should encourage them to do so. They may need further support to help them deal with what they've experienced. Other support can be offered by health professionals in your setting or by calling NHS 111.*
> *You can call 101 on their behalf and with their permission pass on their details.*
> *You can call 101 after the person has left and explain the situation without giving details. If the police consider this a vital piece of information they may then get back in touch and follow normal Police and NHS processes to access this information in a safe and secure way.*

Source: Email communication to safeguarding teams of 23 May 2017

NHS staff are here tasked with performing tasks to assist with Police investigations. There are considerable parallels here to the information sharing undertaken between NHS Digital and the Home Office to detect undocumented migrants, recently overturned by the Health Select Committee.[40]

The overlap between policing and health also stretches to counterterrorism briefings. Each NHS trust receives a daily counter-terrorism briefing from the Home Office. These briefings are often exceptionally long, detailing every news report on terrorism and counterterrorism in the English language from across the globe.

[39] Hilary Garrett – Director of Nursing, NHS England (2013) Prevent Letter for Commissioner Organisations, 18th September 2013.
[40] Dr Sarah Wollaston, 'Letter to Sarah Wilkinson, Chief Executive, NHS Digital, regarding sharing of patient address information with the Home Office for immigration enforcement processes', 23rd January 2018 (London: Health Committee).

OFFICIAL

Monday 24th July Counter-Terrorism Media Summary

This media summary is not a statement of Home Office policy or opinion. It is intended for private use and should not be forwarded outside of your department.

Executive Summary

Leading CT Stories:

- In breaking news this morning, there has been widespread UK and international media coverage of news of a suicide car bombing in the Afghan capital, Kabul, which has killed at least 35 people and wounded 40, doubling earlier casualty estimates. Afghan police said earlier this morning that a suicide attacker had detonated a car bomb in the western part of Kabul, but Reuters reports that, as yet, the target of the attack remains unclear.

The Counter-Terrorism Media Summary is sent to Heads of Security in the NHS, but they can forward these briefings to safeguarding teams. It is unclear why the NHS would require daily briefings on international terrorism for its work but the Home Office clearly considers it a relevant partner in providing security. This contextualises the performance of the Prevent Duty in this NHS.

Safeguarding Teams also receive direct communications from NHS England called 'National Prevent Updates'. These often cover the changing arrangements for Regional Prevent Coordinator support and include reminders for submitting Prevent referral data to NHS England. They also address 'negative' coverage of Prevent in the media and offer counter-narratives to Safeguarding Teams, even if this media coverage is based on academic research; for example:

1. Anti-Prevent messages and counter arguments

You may well have seen that Prevent has again been receiving some bad press. In particular the Open Justice Society report entitled 'Eroding Trust'.

The Home Office have circulated the below which you may wish to use in countering these negative, misinformed and ill-judged media sound bites.

Source: NHS England National Prevent Update – Number 5, 20th October 2016

The communication went on to highlight perceived flaws in the Open Justice Society report and provided a counter-narrative to claims that Prevent is 'police-led' and that information governance of Prevent referrals is lacking.

The tone of these communications raises concerns about the integration of safeguarding teams in a context that extends beyond adult protection. They are also briefed by Counter-Terrorism Units about the characteristics of banned or potentially extremist groups. These briefings detail the rise in Kurdish activism, through to summaries of the National Front, and locations of mosques hosting meetings with suspected links to Hizb-ut-Tahrir:

BACKGROUND

Hizb Ut-Tahrir is an international political organisation which describes its ideology as Islam. Its aim is to re-establish the Islamic Caliphate or Islamic State and to unify the Muslim community worldwide and enforce Sharia law. HuT do not necessarily want to enforce Sharia Law in the UK but to unify all Muslim countries or Islamic lands as they refer to them.

They are a non-proscribed group in the UK but remain of interest both locally and nationally due to their secretive nature and rhetoric.

- **Pakistani Community Centre, Jervaulx Crescent, Bradford**

From looking at the Bradford Dawah Carriers Face Book page it is evident that the Pakistani Community Centre in Bradford has previously hosted events for them. However it is not clear if the community centre were aware of this at the time or are indeed HuT supporters / advocates themselves. They could well be simply renting their space to groups to use.

From the official HuT website www.hizb.org.uk it was found that in July 2014 the community centre allowed Bradford Dawah Carriers to host a meeting entitled "GAZA Emergency Community Meeting". Also in February 2011 HuT actually held a meeting here for the Bradford Muslim community to discuss the then current uprisings in Tunisia and Egypt.

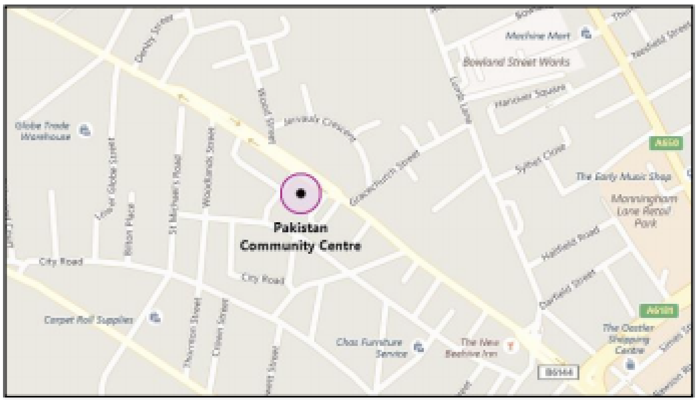

Map of Pakistani Community Centre

Source: 'Who are Hizb-ut-Tahrir', NHS England Safeguarding Folder, August 2017.

NATIONAL FRONT
**IDEOLOGY:** WHITE NATIONALISM, BRITISH NATIONALISM, BRITISH FASCISM<br>**MEMBERSHIP:** BETWEEN 200-300 MEMBERS<br>**ACTIVITY:** STREET ACTIVITY SUCH AS MARCHES AND LEAFLETING. SINCE REFORMING IN 2010 ACTIVITY HAS SHIFTED TO ELECTORAL CAMPAIGNING. RECENTLY CELEBRATED ITS 50TH ANNIVERSARY.<br>**ONLINE ACTIVISM:** WORK CLOSELY WITH OTHER NEO-NAZI WEBSITES. NATIONAL FRONT OFFICAL MAGAZINE/PUBLICATION IS CALLED THE FLAME

Source: 'Extreme Right Wing', NHS England Safeguarding Folder, August 2017

In summary, the communications received by Safeguarding Teams provide important contextual information about the situation of Prevent in the NHS. Briefings from NHS England, the Home Office and Counter-Terrorism Units emphasise the counter-terrorism and policing tasks shared with safeguarding teams. Beyond Prevent, safeguarding teams are encouraged to perceive their role in terms of crime prevention and detection, as well as the safeguarding of people with care and support needs.

Our investigation then moved away from safeguarding teams to explore the effects of Prevent training on NHS staff.

Part 6:

How does staff training frame Prevent as Safeguarding?

How does staff training frame Prevent as Safeguarding?

The Home Office produced DVD 'Workshop to Raise Awareness of Prevent' (WRAP) is used to train NHS staff to spot the signs of radicalization and make referrals if they notice that 'someone may be becoming involved in or supporting terrorism.'[41]

WRAP is shown within the range of mandatory training sessions for healthcare professionals, such as courses on hand washing, fire safety, or infection control.

WRAP can be delivered without any formal training. Anyone who has themselves attended a WRAP session and is 'confident in answering questions that sometimes arise'[42] can deliver the product. The instructor's role is to read from the facilitator's script,[43] play two case studies from the DVD and lead the group discussion after each case study.

The DVD has six parts: 'Introduction', 'Susceptabilities' (sic), 'The risk of radicalisation,' 'Behaviours,' 'What to do,' and 'Action Plan.' The objective is to develop a 'basic understanding of Prevent' and on the basis of already existing 'skills and support' in safeguarding, 'bring the Strategy to life and make a difference to vulnerable people.'[44] The training explicitly frames Prevent Duty as safeguarding:

'It's safeguarding. A means to support vulnerable individuals who may be at risk, in this case, of being radicalized. And if we can stop that, then we genuinely might prevent terrorism in fact all sorts of serious crimes from being committed.' [45]

The section on 'Susceptabilities' [sic] offers several case studies which focus on different ideologies, featuring someone who has been safeguarded under Prevent. The trainer can choose from the following options which are narrated in the voice of the individual who received intervention: 'Adolescent Far Right Extremism,' 'Adolescent Al Qa'ida influence,' 'Adult Far Right Extremism,' Adult Al Qa'ida influence 1', and Adult Al Qa'ida influence 2.'

The Facilitator's Handbook explicitly primes the trainer to ward off audience suspicions that Prevent isn't safeguarding. The handbook instructs them to counter any perception that Prevent's framing as safeguarding is 'too much of a leap' with the argument that **our perceptions of terrorism are 'too narrow' if we think in this way:**

> The link into this piece from the video simple; say what safeguarding means to you, and how a leap to terrorism from that might seem like a bit of stretch. Say you found it a stretch – put yourself in their shoes. Then say the reason you used to find it so was because your view of terrorism was too narrow…
>
> Remind the audience this not only about preventing people carrying out the attacks but supporting those attacks – and illustrate this by showing a rise up the iceberg towards breaking through the waterline.
>
> 1. Prevent = safeguarding. Too much of a leap?
> 2. If yes, because our view of terrorism too narrow

Source: Home Office (undated) Workshop to Raise Awareness of Prevent: Facilitator's Handbook (London: HM Government), p.4.

[41] *Home Office (2015) Workshop to Raise Awareness of Prevent, Workshop Script (London: HM Government), p.1.*
[42] *NHS England (2016) 'NHS England National Prevent Update Number 2', August 2016, pp.2-3.*
[43] *Prevent Leads and Safeguarding Experts unanimously told us that they do not stick to the script, contra instructions, because it becomes impossible to retain the audience's attention.*
[44] *Home Office, Workshop Script, p.1*
[45] *Home Office (2015) WRAP: Workshop to Raise Awareness of Prevent, Training Video (London: HM Government)*

The script also makes clear that participants 'don't need to understand the ideologies or ideas that are promoted' and instead, in the group exercise they are encouraged to identify 'vulnerabilities' or 'susceptibilities' by relating to people's 'emotions and feelings' since 'these are after all what are truly exploited by the radicaliser.'[46] The question driving the discussion is 'What factors could make someone susceptible or vulnerable to carrying out or supporting violent, criminal or terrorist acts?' and trainers are instructed to work with the group in the following way:

How to run the Vulnerabilities exercise

- Keep turning the circumstantial reasons given for vulnerability into emotions or feelings where you can. This will mean you are far more likely to solicit key words such as Isolation; Belonging; Need; Anger; Desire; Frustration; Grievance…
- Isolation or Exclusion from the mainstream are clearly important factors in allowing someone to be radicalised so its great if these can make an appearance (all the case studies allow for this quite naturally)
- Use this exercise to work around the room – don't do it as a free-for-all shout out. The reason for this is when it comes to the second exercise, if the audience don't believe you might point to them and ask for an answer some might opt out. Keep them on their toes and let them think you might ask for their opinion or input at any given time.

Source: Home Office (undated) *Workshop to Raise Awareness of Prevent: Facilitator's Handbook* (London: HM Government), p.6.

When it comes to spotting emotional states that are associated with radicalisation in the training, the Facilitator's workbook gives the following instruction:

Emotional behaviours aren't easy to articulate – really challenge them on this; they know the answers! Crying, quick to temper, mood swings, sullen, their demeanour – these are all good answers. You know when someone is down (or up for that matter); stressed; preoccupied; conflicted; so this allows us to just take a few minutes and really think about what that looks like.[47]

Sample answers are then provided to link the emotional state to potential problem behaviours:

EMOTIONAL	VERBAL	PHYSICAL / CIRCUMSTANTIAL
Short tempered	Fixated on a subject	Tattoos
Angry	Closed to new ideas/conversations	Use of internet
New-found arrogance	Change in language/use of words	Change of routine
Withdrawn	Asking inappropriate questions	New circle of friends
Depressed	"Scripted" speech	Absent
Crying	Saying inappropriate things – a call to violent action	Letting themselves go (in terms of appearance)

Source: Ibid, p.16

[46] *Home Office (undated), Facilitator's Workbook, p.6.*
[47] *Home Office, Facilitator's Workbook, p.6.*

The 'What to do' section starts with the second case study narrated by a professional who made a referral in the context of education, higher education, health, local authority, community, and offender management. After the identification of behaviours of concern, this is a call to 'do something.' The 'Notice Check Share approach' is revealed in the 'Action Plan' section that describes the process of making a referral to the Channel Panel. The training video concludes by summarizing the main message of the training:

> *'These are individuals who are at risk. So I personally would far rather hear about them now than see them hit the headlines at a later date […] We can all have the confidence to trust our instincts and speak up when someone needs our help.'*[48]

WRAP training is focused on using instincts or gut feelings in detecting signs of radicalisation and making referrals on the basis of them. It builds on forms of professional intuition developed in safeguarding practice by introducing an idea of radicalization as grooming. The training, delivered by a non-expert facilitator, does not impart expertise or factual knowledge about recruitment into terrorism or the distinction between extremist and non-extremist ideology. Indeed the Facilitator's Script is sometimes refreshingly honest about the effectiveness of the training:

> *For most or all of you you'd be a Zero if we were ranking Prevent knowledge out of 10. And my job is just to take you to 1 or 2 on that scale with this workshop.*[49]

Having analysed the pedagogy of the WRAP product, our research questions became

> *Are recipients comfortable with WRAP and confident to make Prevent referrals? And, given WRAP's instruction to follow their gut instincts, what would they report?*

[48] *Home Office (2015) WRAP: Workshop to Raise Awareness of Prevent, Training Video (London: HM Government)*
[49] *Home Office, Full Workshop Script, 1.*

Part 7:

Survey with NHS Staff on WRAP training and the Signs of Radicalisation

Survey with NHS Staff on WRAP training and the Signs of Radicalisation

We conducted an online survey on NHS staff attitudes to the Prevent Duty and the hypothetical scenarios they would report as a Prevent concern. 329 respondents answered the following questions:

1. "Please grade how well the Prevent training explained the signs of radicalisation"

STRONG CONFIDENCE

Overall, **staff confidence in Prevent training is high across the Midlands region.** 2 respondents (<1%) thought that their training was 'extremely bad' at explaining the signs of radicalisation. 3% thought that their training explained the signs 'badly'; 25% answered within the range provided for 'Okay'; 47% answered 'well'; and 24% 'extremely well'. **WRAP's focus on intuition and gut feelings is interpreted as clearly explaining the signs of radicalisation.**

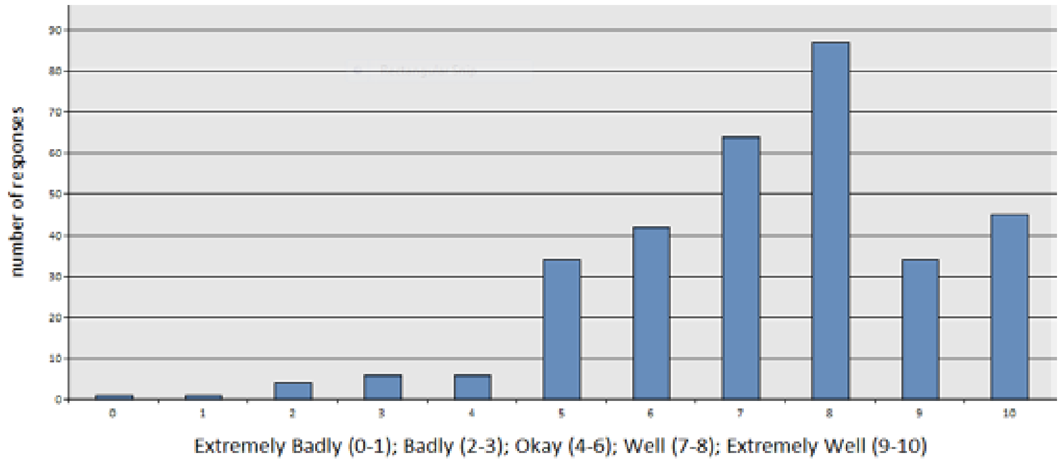

2. "Having attended Prevent training, how confident would you feel in referring a query about radicalisation?"

CONFIDENT

We asked survey respondents how confident they would be to make a Prevent referral after receiving their training. Across the region, results were more evenly distributed than for 'grading the training on explaining signs of radicalisation'. **The results show more balance between 'confident' and 'neither confident nor unconfident'.** 2% of regional respondents stated they were 'extremely unconfident' to make a referral after their training; 5% were 'unconfident'; 34% were 'neither confident nor unconfident'; 39% were confident; and slightly over 19% were 'extremely confident'.

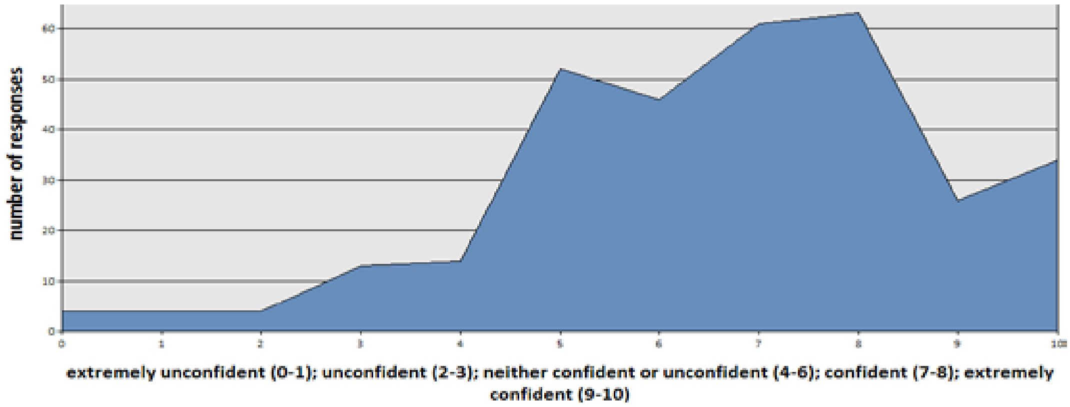

3. "Please grade how comfortable, or uncomfortable, you felt with the material presented during Prevent training"

STRONG COMFORT

We then asked people to grade their comfort with the material presented during their Prevent training. **Regionally, results were strong in this category – almost three quarters of respondents had positive attitudes towards the material.** 1% were 'extremely uncomfortable'; 2% were 'uncomfortable'; 25% were 'neither comfortable nor uncomfortable'; 35% were 'comfortable'; and 37% were 'extremely comfortable'.

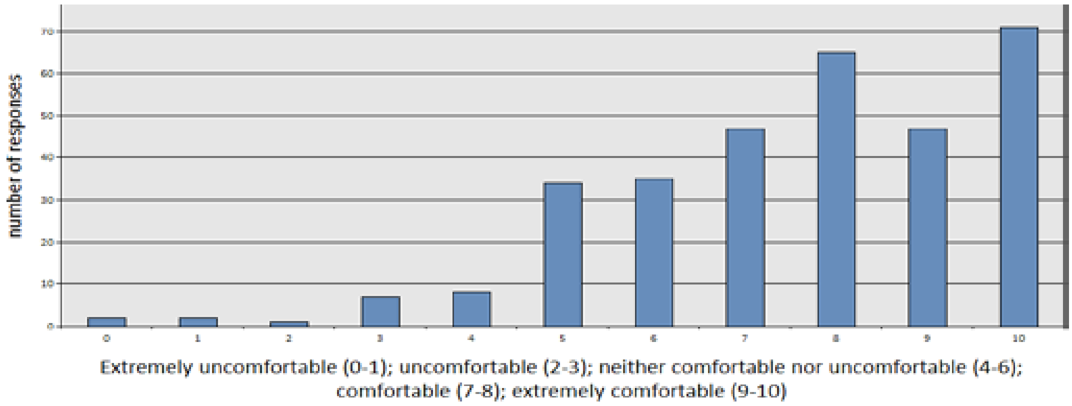

At first glance, these findings tend to support the conclusions of a study which explores the roll-out of Prevent in education; specifically that the majority of public sector respondents accept the core government narrative that Prevent is a safeguarding duty.[50] **However, when we directly asked healthcare professionals whether they understood the Prevent Duty to be a form of safeguarding, our results became far more ambiguous. Only 47% reported agreement with the statement that "Prevent is just safeguarding.** It is the same as safeguarding people from domestic abuse, financial abuse and sexual abuse". Slightly over 30% of people 'didn't know' whether Prevent fits the profile of safeguarding, whereas 22% disagreed with the statement:

4: "Prevent is just safeguarding. It is the same as safeguarding people from domestic abuse, financial abuse and sexual abuse"

SPLIT DECISION

This suggests that the context of *safeguarding in health affects respondents' attitudes towards the Duty*. In healthcare, unlike education, safeguarding is associated with protective intervention upon adults with 'care and support needs'. Our results show that the majority of healthcare professionals (in our study) view Prevent training positively; however this acceptance *does not signify a similar acceptance that Prevent is safeguarding.* Neither does lead to an unquestioning acceptance of the place of Prevent in healthcare:

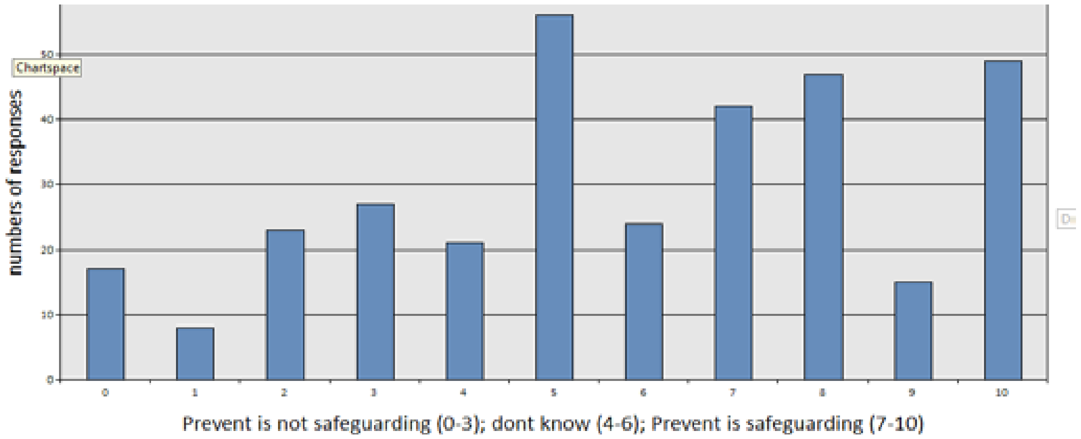

[50] Busher, Joel; Tufyal Choudhury; Paul Thomas & Gareth Harris (2017) *What the Prevent duty means for schools and colleges in England: An analysis of educationalists' experiences*, Report available from: http://eprints.hud.ac.uk/id/eprint/32349/

5: "The Prevent Duty belongs within healthcare"

SPLIT DECISION

Regionally, opinion on whether Prevent belongs within healthcare is quite polarised. In total, 48% express an attitude that it does belong (agree + strongly agree). **52% don't know, or disagree, that Prevent belongs in healthcare.**[51]

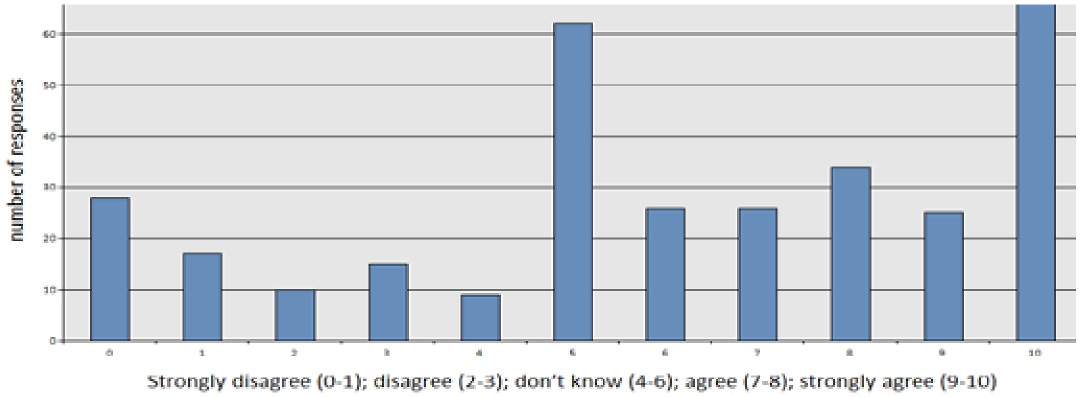

But this polarisation of opinion does not translate to mistrust in individual organisations, rather it is directed at the Prevent Duty at the national level. Respondents have strong confidence in their Trusts and CCG's to make appropriate Prevent referrals.

6: "I trust my organisation to make sensible and appropriate decisions about referring people to the Local Authority through the Prevent Duty"

STRONG CONFIDENCE

4% of respondents expressed negative views about their organisation's capacity to make sensible and appropriate decisions on Prevent referrals. 21% 'didn't know'; and 75% of respondents expressed 'strong' or 'milder' degrees of confidence.

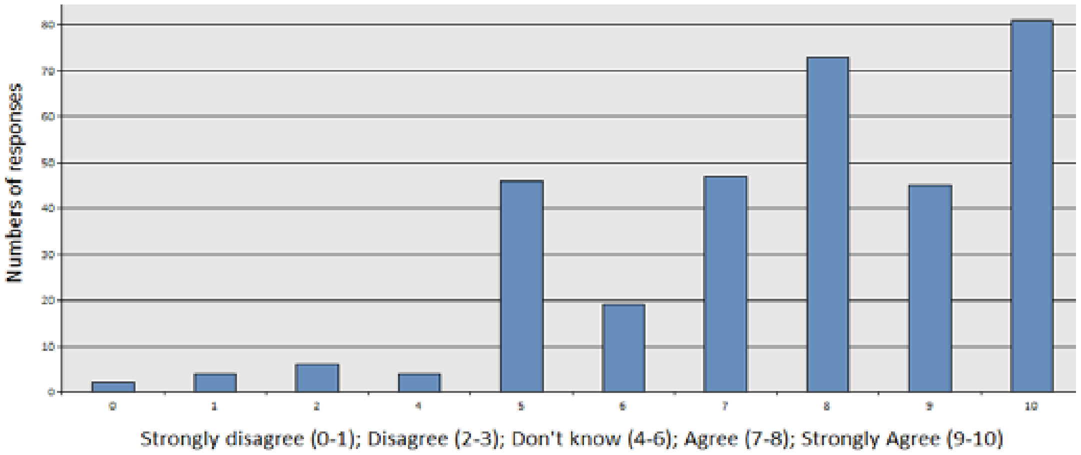

[51] *In detail: 14% strongly disagree that Prevent belongs in healthcare; 8% disagree; 30% don't know; 19% agree Prevent belongs in healthcare; and 29% strongly agree.*

7: "The Prevent Duty is a form of surveillance"

SPLIT DECISION

We then turned the tables to explore the details of objections to Prevent in healthcare. **Interestingly, most staff in the region are unsure if Prevent is a form of surveillance. This matches the high 'don't know' scores for figure 4, 5 & 6.** 33% say that Prevent isn't surveillance; 43% aren't sure; and 24% think that the Prevent Duty is a form of surveillance.

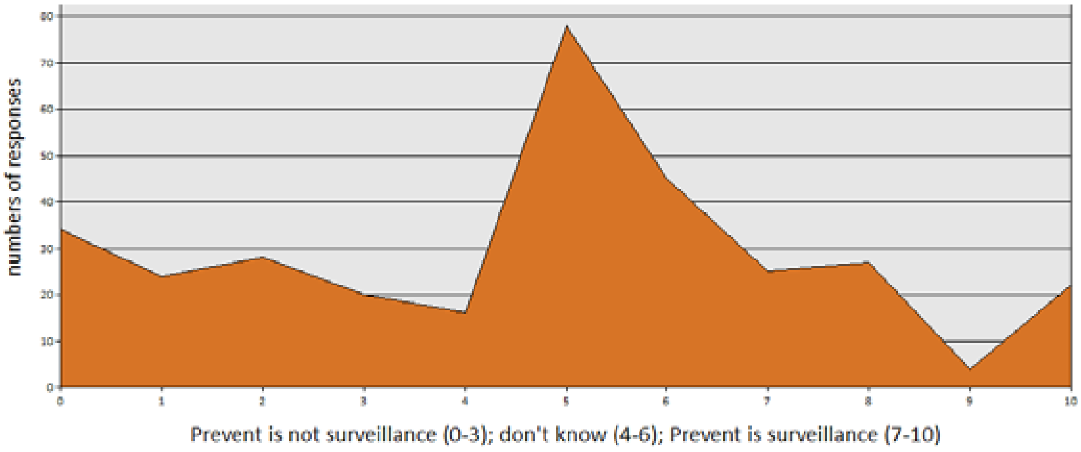

Given the emphasis on intuition when noticing radicalisation concerns, what behaviours do staff understand as radicalisation indicators?

8: "If a patient or staff member possessed books about radical Islamic philosophers or radical anarchism, how likely is it that you would make a safeguarding query?"

STRONG ASSOCIATION

We deliberately didn't clarify the distinction between radical and non-radical theory in this question, to see how people would respond. Only one respondent (from 329) commented that they doubted their ability to distinguish radical Islamic/Anarchist philosophy from non-radical. As such, **over 99% of respondents feel confident to distinguish radical books from non-radical ones – without training in this area.** Having trained in this area, the Primary Investigator advises that postgraduate study in Terrorism Studies or Theology would be required to enable someone to make such a distinction. As such, WRAP could make NHS staff over-confident in their abilities to detect extremism.

The possession of radical philosophy was thought to be a very significant indicator of radicalisation (rather than a sign of general reading or educational study). **In total, 70% of respondents were 'likely', or 'very likely', to make a Prevent query on the basis of possession of Islamic/Anarchist philosophy books**.[52]

[52] 9% said that they 'don't know' if they would report possession of radical philosophy; less than 7% said that it would be 'extremely unlikely'; 15% answered 'not likely'; 38% answered 'somewhat likely'; and 32% said 'very likely'.

[53] Coolsaet, Rik (2015) What Drives Europeans to Syria, and to IS? Insights from the Belgian Case, Egmont Paper 75, March 2015; Roy, Olivier (2011) 'Al Qaeda: A True Global Movement' in Jihadi Terrorism and the Radicalisation Challenge: European and American Experiences, edited by Rik Coolsaet (Abingdon: Routledge), pp.19-26.

Academic research suggests that the philosophically and religiously literate are actually less susceptible to radicalisation,[53] and WRAP training makes no mention of philosophy books as an indicator of concern, so we believe that r**espondents are drawing their attitudes from popular culture rather than official training or academic research.**

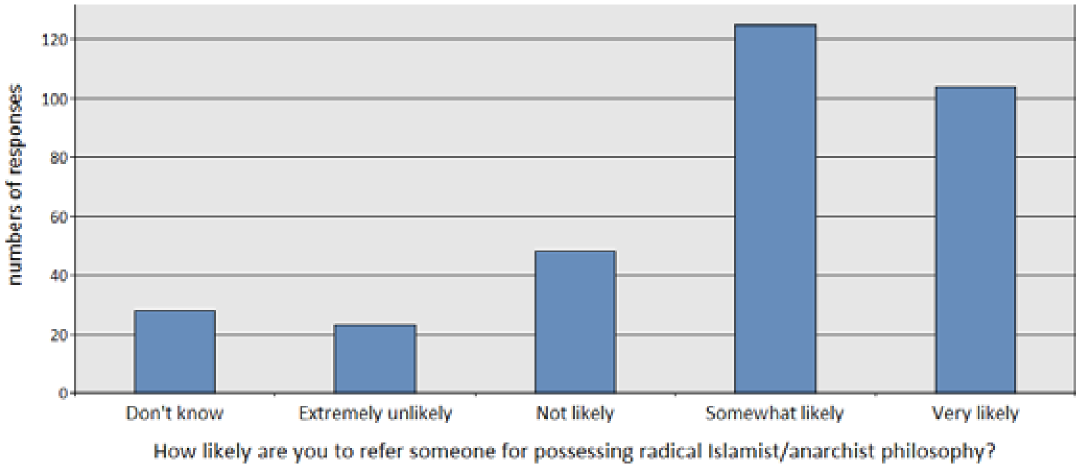

9: "If you saw a patient or staff member watching video clips of beheadings, would you make a safeguarding query?"

STRONG ASSOCIATION

This is confirmed by respondents association of beheading videos with signs of concern. Beheading videos are not mentioned in Prevent training, so the association of viewing such videos with radicalisation comes from popular media. When asked if they would make a Prevent query about someone who watched beheading videos, 74% of respondents said 'yes' (without any reference to the 'care and support needs' generally needed for a concern to become relevant to safeguarding procedures). 21% 'didn't know' and only 5% said they wouldn't make a Prevent referral on this basis.

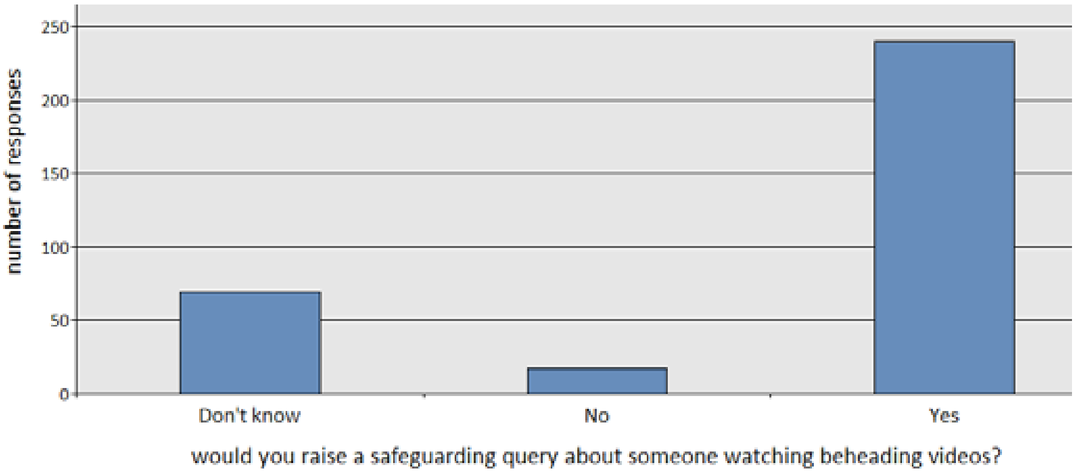

10: "If a patient or staff member made hateful statements against an ethnicity, sexuality, or another minority group, how likely is it that you would make a safeguarding query?"

STRONG ASSOCIATION

Other criteria which healthcare staff strongly associated with radicalisation included hate speech about ethnicities, sexualities and other minority groups. An enormous 82% of respondents reported that they would be 'very' or 'somewhat' likely to make a Prevent query upon hearing such hate speech. Only 15% were 'not likely' or 'very unlikely' to make such a referral. **While it is important to challenge hate speech in the workplace, the Prevent training does not specify that illiberal opinion indicates radicalisation.**

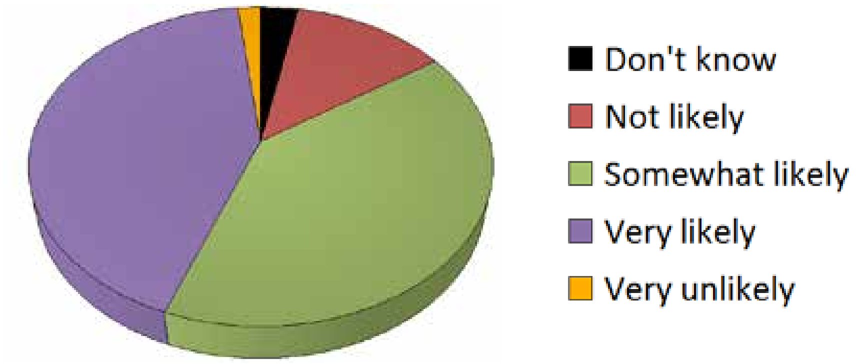

11: "Grade how confident you feel to tell the difference between someone experiencing radicalisation and someone with an interest in Middle Eastern politics and wars"

LOW STAFF CONFIDENCE

One of our most significant findings was that healthcare professionals' confidence in their ability to spot radicalisation fell away when we introduced mildly complex scenarios. For example, we asked respondents to grade their confidence in distinguishing radicalisation from someone's interest in Middle Eastern politics and wars. **Only 1 in 3 respondents reported having any degree of confidence that they could make the distinction.** 56% of participants stated that they 'didn't know' if they could distinguish interest in Middle Eastern politics and wars from radicalisation, and 11% were explicitly unconfident to tell the difference:

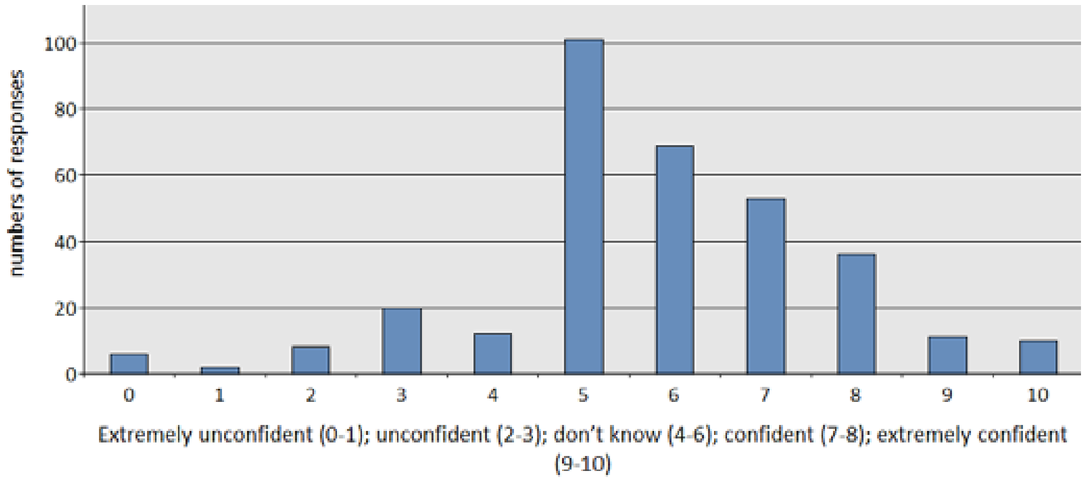

12: If you repeatedly heard a patient or staff member express anger about British and American wars overseas, how likely is it that you would you make a safeguarding query?

SPLIT DECISION

We asked this question to measure the extent to which people associate radicalisation with anger about foreign policy. Responses were split down the middle. 6% were 'extremely unlikely' to make a Prevent query on the grounds of foreign policy related opinion; 41% were 'unlikely to'; 30% were somewhat likely to make the query; and 13% were 'extremely likely'.   Slightly over 9% answered that they didn't know.

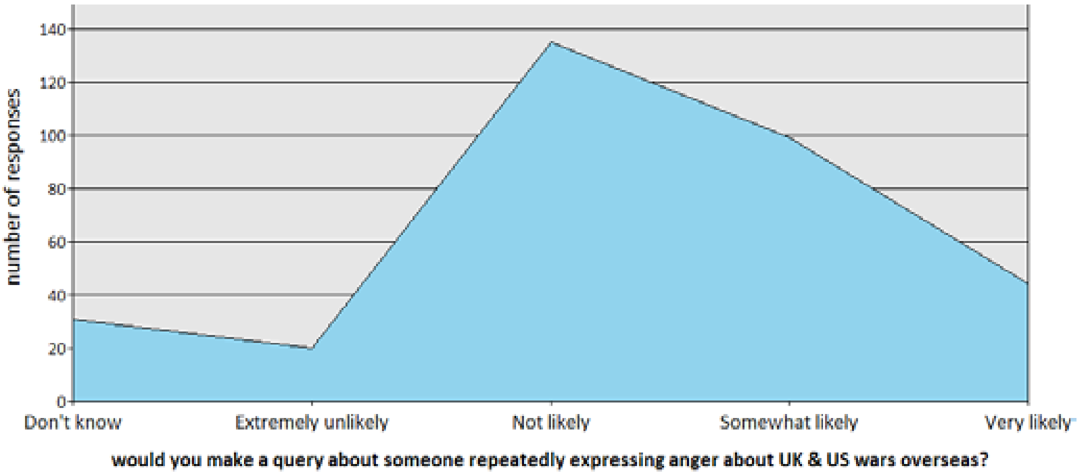

13: "If a patient or staff member made violent threats against the British Armed forces, how likely is it that you would you make a safeguarding query?"

STRONG ASSOCIATION

Finally, given the successful and failed terrorist plots against British soldiers in recent years, we wanted to test the extent to which people connect threats against the armed forces to radicalisation. The results showed that people do draw their knowledge of radicalisation indicators from the news about terrorist plots. Regionally, there was **an extremely strong association between 'threats being made against the Armed Forces' and people's willingness to make a Prevent referral.** 89% of people were 'very likely' or 'somewhat likely' to refer on those grounds. Only 6% of people were 'unlikely' or 'extremely unlikely' to refer a person making such threats. 5% answered that they didn't know.

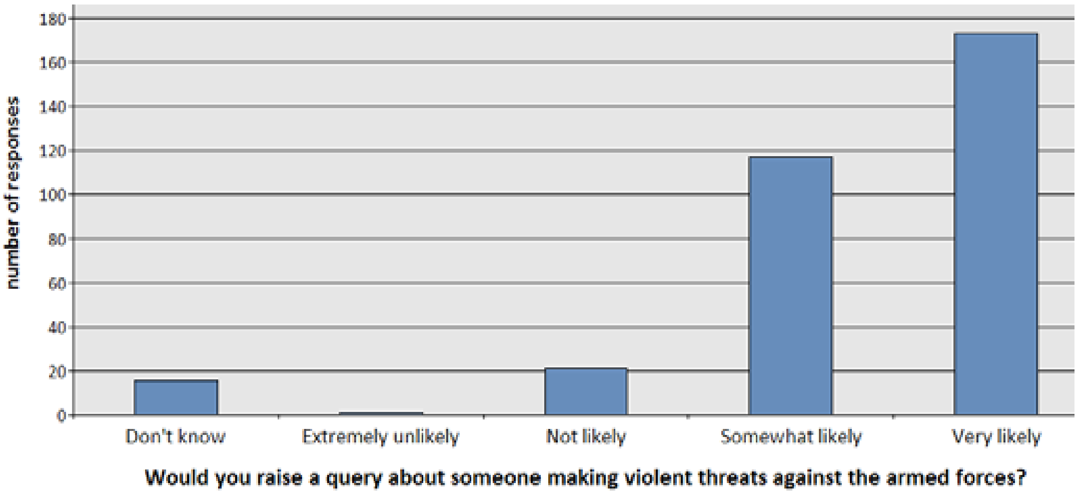

Survey Conclusions

- Staff confidence in the Prevent training and comfort with the material is high.
- But - extremely high numbers of NHS staff associate the possession of radical Islamic/anarchist philosophy with radicalisation, rather than with education or general interest.
- Extremely high numbers of NHS staff associate radicalisation with hate speech against ethnicities, sexualities and other minorities. Illiberal speech is being associated with radicalisation and staff would make Prevent queries on the basis of hate speech.
- Only 1 in 3 respondents expressed any confidence about knowing the difference between political interest in the Middle East and radicalisation – **an important reflection on the gaps in WRAP training.**
- High numbers of NHS staff would make Prevent queries about people who watch beheading videos. As such, **many NHS staff draw their understanding of radicalisation indicators from popular culture representations.**
- Extremely high numbers of NHS staff associate 'violent threats against the Armed Forces' with radicalisation.

Discussion of Survey Findings

Illiberal attitudes, beheading videos and philosophy possession as interpreted by respondents as pre-stages in terrorism - but possessing unpleasant opinions, or reading religious philosophy are not indications of radicalisation. NHS England has emphasised this in the most recent policy guidance for implementing the Prevent Duty:

> *Professionals should also have due regard to the Public Sector Equality Duty and be sensitive in their considerations. Outward expressions of faith or an interest in global or political events, or opinions that may seem unpleasant, in the absence of any other indicator of vulnerability or risk are not reason to make a referral to Channel.*[54]

And yet, our study shows that NHS healthcare professionals are inclined to make Prevent referrals on these grounds. On the basis of our survey, questions about the effectiveness of Prevent training can be raised. Research should be conducted at the national level to further test the scenarios NHS staff associate with radicalisation after receiving WRAP training. Invoking 'gut feeling' and intuition as the bases upon which staff should make referrals is unscientific and our survey shows that staff are inclined to use popular culture representations as guides to radicalisation. This reliance on 'intuition' also risks allowing unconscious bias to influence referrals.

[54] *NHS England (2017) Guidance for mental health services in exercising duties to safeguard people from the risk of radicalisation (London: HM Government).*

However, when we posed the question of inappropriate referrals to safeguarding experts and police dealing with referrals, they were unconcerned. Interviewees felt that misguided referrals were unproblematic because multiple levels of triaging are built into the Prevent referral system:

Interviewer:
"but can intuition be trusted, I guess, is my question?"

CCG Safeguarding Expert C:
"Well, it doesn't have to be, because somebody else will test it for you. *You've got a whole triage process who will look at somebody's presentation, their circumstances, their background, matching that against a set of criteria in Safeguarding Adults. So you don't need to. That's what I say to GPs, you know, we're not looking at gold standard diagnosis here; we're not looking at a matrix of eight-by-eight. What we're looking for is a little bit of concern about the way somebody's behaving, presenting or, you know, or speaking. If you have that doubt, then pass that on, and somebody else will deal with it."*

Essentially this argument suggests that misguided referrals are unproblematic because a triaging process removes them from the system, before the subject finds out they have been referred. Our interview with a Police Prevent Lead confirmed this perspective, by suggesting that the Police don't want to discourage ***any*** referral – because the importance of catching a real case of radicalisation far outweighs the problems of misguided referrals:

"We'd rather the referral comes in, and we can check there's nothing more to it, than doesn't come in. *We want people to be confident to refer. We want to avoid missed opportunities. Like the man who wanted to bomb the Bristol shopping centre, he reported to hospital with burned hands and feet because he was experimenting making a bomb which went off. That was a missed opportunity.* ***We'd rather that these things were reported, even if they don't end up being relevant to Prevent."***

This is not a safeguarding rationale. The encouragement of referrals which might not end up being Prevent-relevant is justified on the basis of 'checking, just in case'. **This is a rationale more familiar to surveillance,** where the behaviours of the population are watched in order to detect instances of crime.

Referrals are maximised so that the Police can check there is nothing sinister occurring. **However, the Prevent referral process does subsequently act like a pathway into societal forms of support.** Home Office statistics for the year 2015-16 show that 50% of people formally referred to Prevent[55] receive mental health treatment, education and housing as a result.[56] Only 5% of formal referrals are allocated to Channel mentor who works with the person to reintegrate them within society.

The Channel process has become further embedded in the provision of mental health treatment to Prevent referrals, with the formalisation of a national pathway between Channel and Mental Health services in 2017.[57] Previously this Urgent Care Pathway was piloted in London, Manchester and Birmingham to accelerate the provision of mental health treatment to those deemed at risk of radicalisation – removing them from Channel. In the words of a forensic psychiatrist leading one of those pilots:

> *I sort of conceptualise the [system] as more like a liaison and diversion scheme really for Prevent cases […]there's people who have terrorist views and a mental health problem and the two are absolutely [linked]. So they've got a delusion related to terrorism, you treat that, it all goes away. So that's one end of the spectrum. The other end is where they have a mental health problem and ideology and they're not related at all. And then there's this big group in the middle. And so we want to try and delineate that a bit and it's going to be a bit Mickey Mouse […] as I say, I'm ashamed, it's totally unscientific and it's going to be based on opinion, so it's a bit crap really, but it's just trying to get at what's going on.*[58]

---

[55] *Where their case is discussed at Channel Panel and not previously removed as being irrelevant or misguided.*
[56] *Home Office (2017) Individuals Referred to and Supported Through the Prevent Programme, April 2015 to March 2016 (London: HM Government).*
[57] *NHS England (2017) Guidance for mental health services in exercising duties to safeguard people from the risk of radicalisation (London: HM Government).*
[58] *Interview with Forensic Psychiatrist A.*

It is always admirable for a society to provide services to those in need. But several questions need to be asked about the formalisation of an Urgent Care Pathway between Prevent and Mental Health.

Firstly, several interviewees mentioned that the context of austerity in public services will mean that these pathways are interpreted as opportunities. With access to social services and mental health provision reduced across society, formalising such a pathway **creates incentives for practitioners to refer patients to Prevent,** hoping that Channel will allocate resources to the service user in need. As one GP told us:

"I know how difficult it is with housing and one of my patients, single man, was made homeless. He would have no chance whatsoever of getting housing. He was referred to Prevent because when he turned up at the housing centre, he was saying, "I want to kill Muslims and Africans". His landlord was an African Muslim. So, he got referred straightaway to Prevent. They got in contact with the Salvation Army. He was housed in a couple of days […] ***There are times where I've actually thought to myself, oh, you know what, yes, you've got housing issues, maybe I should say they're a danger to society, they're at risk of radicalising."*** [59]

Similarly, CCG Safeguarding Expert C told us that this redirection of resources to a small contingent of vulnerable people, and away from others, was a big concern for multi-agency panels about Prevent. Similarly the interviewee alluded to the incentives this creates:

Young people get crap poor mental health services […] I used to work with kids who used to go into care, you'd get a free pair of new trainers, not cheap ones either; you got Nikes or Puma. Kids were sussed, you know! And you get people into Safeguarding, you get resources. You get people into Prevent. One of the criticisms that does come out of this multi-agency panel is that this person goes into Channel, yet you can't provide care for older people in the community. Yet there's all the resources going into this one person! ***And that's the difficult bit, that's the uncomfortable bit of the Prevent that they say "how come they get all that, when we're trying to get Mrs Jones home care for the past six months"?***

[59] *Interview with GP 1, 18th September 2017.*

If GPs, Safeguarding Experts and Multi-Agency Safeguarding panels note problems surrounding the redirection of scarce resources, and the incentives this creates, further research is needed into how this is affecting Prevent referrals made by public sector workers.

Secondly, one must consider the logic of shaping Prevent as a pathway through which public services and support are administered. The NHS will now not only make Prevent referrals about patients – it will also receive those Prevent cases and provide them with mental health support. **Does the NHS receive the same people back, which it originally referred to Prevent? If so, why not refer people directly to Mental Health in the first place?**

Direct referrals to mental health services, or social services, would be more effective and it would avoid the verification (and permanent recording) of the referral by specialist police.

What happened to people referred through Prevent

Figures for 2015-16.

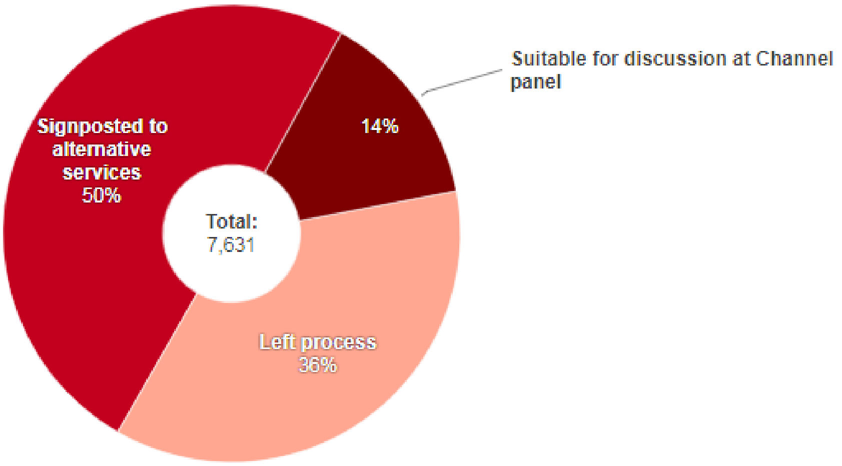

Source: Home Office

This circularity in the Prevent process is connected to widespread societal anxiety about terrorism, rather than the most effective way of directing people to the services they require.

Finally, there is evidence to suggest that the mentally ill are being inappropriately stigmatised as terrorism risks as a result of policy framings of vulnerability factors.

Part 8:

Radicalisation Screening, Prevent and Mental Health Trusts

Radicalisation Screening, Prevent and Mental Health Trusts

Since November 2017, there has been separate guidance for Mental Health Trusts on Prevent. Mental Health is positioned as both a referring institution into Prevent, and a recipient of people referred to Prevent.

NHS England Prevent Guidance for Mental Health Trusts relies upon the research of Emily Corner, Paul Gill and Oliver Mason, which argues that up to 40% of lone actor terrorists have some form of mental illness.[60] Indeed, that is the only academic study used to support the Prevent Strategy for Mental Health. NHS National Medical Director Sir Bruce Keogh also invoked the same authors when outlining the importance of the Mental Health sector to Prevent, in a letter sent to all chief executives and medical directors of mental health trusts.[61] But is important to recognise that *the authors of the study do not consider mental illness to be a predictor of lone actor terrorism*; rather it is a correlate.

Furthermore, the extent of the correlation should be taken with a pinch of salt. The dataset used by Corner & Gill is developed from one used in 'Bombing Alone: Tracing the Motivations and Antecedent Behaviors of Lone-Actor Terrorists' by Paul Gill, John Horgan and Paige Deckert.[62] **The authors never analysed or met any terrorists. Instead their dataset was created through searches of news media for lone actor terrorists, after 1990, and media descriptions that they suffered from poor mental health.** The dataset is comprised of only 119 offenders and only includes terrorist events reported in the media.

Academically, one can have serious reservations about the reliability of the dataset underpinning claims within NHS England Guidance for Mental Health services around Prevent. This is particularly worrying because the framing of mental illness as a vulnerability for radicalisation in the Prevent Strategy is having effects. **Sir Bruce Keogh confirms that two-thirds of NHS referrals to Prevent come from Mental Health Trusts.**[63]

To investigate this pattern, we sent FOI requests to all Mental Health Trusts in the UK, asking whether they screen patients for signs of radicalisation.

49 Trusts responded. Most would only apply specialist forensic risk scoring tools to patients if a concern about radicalisation was noted. **However 4 Trusts currently screen all service users for signs of radicalisation as part of their Comprehensive Risk Assessment procedures.** This is despite the Royal College of Psychiatrists' concern about the validity of a link between mental illness and terrorism and 'the conflicts between the duties of a doctor' and Prevent.[64]

In South London and Maudsley, every service user is screened for signs of radicalisation as part of the regular risk assessment process. The checklist for 'radicalisation or exploitation' does not fall within the safeguarding section of the assessment, but stands alone:

[60] *Emily Corner and Paul Gill (2015) 'A False Dichotomy? Mental Illness and Lone-Actor Terrorism," Law and Human Behaviour 39, pp. 23–34; Emily Corner, Paul Gill & Oliver Mason (2016) 'Mental Health Disorders and the Terrorist: A Research Note Probing Selection Effects and Disorder Prevalence', Studies in Conflict & Terrorism, DOI: 10.1080/1057610X.2015.1120099*

[61] *Sir Bruce Keogh (2016) 'Strengthening the Mental Health Response for People at Risk of Radicalisation', letter sent to Chief Executives and Medical Directors of Mental Health Trusts, 15 November 2016.*

[62] *Gill, Horgan and Deckert describe how their dataset was created "by searching academic literature on lone actor terrorism for perpetrator names and doing a LexisNexis news search. More individuals were also identified through the Global Terrorism Database developed by the National Consortium for the Study of Terrorism and Responses to Terrorism (START), and through lists of those convicted of terrorism-related offenses in the United Kingdom and the United States. The decision was then made to limit the population to post-1990 events because a large component of the data would be coded from the LexisNexis archive - which is generally quite sparse before the 1990s. In total,* ***119 lone-actor terrorist offenders fit the specified geographical, temporal, and operational criteria.*** *The authors collected data on demographic and background characteristics and antecedent event behaviors by examining and coding information contained in* ***open-source news reports, sworn affidavits, and when possible, openly available first hand accounts.*** *" See Paul Gill, John Horgan and Paige Deckert (2013) 'Bombing Alone: Tracing the Motivations and Antecedent Behaviors of Lone-Actor Terrorists', Journal of Forensic Sciences 59(2), pp.425-35 (emphasis added).*

[63] *Sir Bruce Keogh (2016) 'Strengthening the Mental Health Response for People at Risk of Radicalisation', letter sent to Chief Executives and Medical Directors of Mental Health Trusts, 15 November 2016.*

[64] *Royal College of Psychiatrists (2016) Counter-Terrorism and Psychiatry: Position Statement PS04/16 (London: Royal College of Psychiatrists).*

[65] *Akimi Scarcella & Ruairi Page &Vivek Furtado (2016) 'Terrorism, Radicalisation, Extremism, Authoritarianism and Fundamentalism: A Systematic Review of the Quality and Psychometric Properties of Assessments', Plos One, https://doi.org/10.1371/journal.pone.0166947*

Radicalisation or Exploitation	Yes	No	Not Sure

History of related Risk Events	
Date	Description of Event
No Historical Risk Events recorded on ePJS	

Risk Factor	Historical	Current	Notes and Comments
**Engagement**			
Exhibiting feelings of injustice and anger			
Being influenced or controlled by a group/gang			
Appears susceptible to extremist indoctrination			
Concerns about online activity			
Impact of mental health issues			
**Intent**			
Over-Identification with an extremist group or ideology			
Demonstrating Them and Us thinking			
Attitudes that appear at odds with values relating to respect for law, democracy, mutual respect, equality?			
Desire to pursue harmful objectives			
**Capability**			
Ability to travel easily (e.g. Passport, money available)			
Knowledge, skills and competencies? (e.g. particular expertise/technical training?)			
Access to networks (online or in person), funding, equipment?			
**Other**			
**Referral**			
**If you haver identified 6 of the points as a current risk, then consider a Prevent Referral**	Yes<br>No		

Source: South London and Maudsley Mental Health Trust (undated)

The standardised screening of those with mental illness for radicalisation inappropriately positions them as a community from which terrorism originates. This particular example even encourages a Prevent referral to be made if the service user meets 6 of the 12 criteria.

As the Royal College of Psychiatrists clearly articulate, there is little evidence to suggest a link between mental illness and terrorism. Furthermore, the evidence base underpinning specific radicalisation and extremism risk scoring tools is heavily criticised in academic literature.[65]

In this scientific climate, the automatic screening of all service users in four mental health trusts for radicalisation and extremism should be postponed until reliable indicators are found.

For more information please contact:
Dr Charlotte Heath-Kelly
c.heath-kelly@warwick.ac.uk

Department of Politics and International Studies
The University of Warwick
Coventry
CV4 7AL
United Kingdom
warwick.ac.uk/fac/soc/pais/research/researchcentres/irs/counterterrorisminthenhs

FUNDED BY

wellcome trust
